# Calcium-Responsive Diguanylate Cyclase CasA Drives Cellulose-Dependent Biofilm Formation and Inhibits Motility in Vibrio fischeri

**DOI:** 10.1128/mBio.02573-21

**Published:** 2021-11-09

**Authors:** Alice H. Tischler, Michael E. Vanek, Natasha Peterson, Karen L. Visick

**Affiliations:** a Department of Microbiology and Immunology, Loyola University Chicago, Maywood, Illinois, USA; University of Connecticut

**Keywords:** diguanylate cyclase, *Vibrio cholerae*, *Vibrio fischeri*, biofilms, calcium sensors, calcium signaling, cellulose, cyclic nucleotides

## Abstract

The marine bacterium Vibrio fischeri colonizes its host, the Hawaiian bobtail squid, in a manner requiring both bacterial biofilm formation and motility. The decision to switch between sessile and motile states is often triggered by environmental signals and regulated by the widespread signaling molecule c-di-GMP. Calcium is an environmental signal previously shown to affect both biofilm formation and motility by V. fischeri. In this study, we investigated the link between calcium and c-di-GMP, determining that calcium increases intracellular c-di-GMP dependent on a specific diguanylate cyclase, calcium-sensing protein A (CasA). CasA is activated by calcium, dependent on residues in an N-terminal sensory domain, and synthesizes c-di-GMP through an enzymatic C-terminal domain. CasA is responsible for calcium-dependent inhibition of motility and activation of cellulose-dependent biofilm formation. Calcium regulates cellulose biofilms at the level of transcription, which also requires the transcription factor VpsR. Finally, the Vibrio cholerae CasA homolog, CdgK, is unable to complement CasA and may be inhibited by calcium. Collectively, these results identify CasA as a calcium-responsive regulator, linking an external signal to internal decisions governing behavior, and shed light on divergence between *Vibrio* spp.

## INTRODUCTION

Bacteria adapt to their surroundings by recognizing environmental signals, resulting in changes in gene expression, protein production and/or activity, and other processes that permit them to survive and/or thrive accordingly. In some cases, adaptation promotes development of biofilms, sessile communities encased within a protective extracellular matrix. Biofilms provide fitness advantages based on social cooperation, resource sharing, and protection from environmental stressors, such as antibiotics (1, 2). Planktonic cells sense and respond to signals by first adhering to surfaces, typically via flagella or other surface structures. Attached cells then produce and secrete the protective matrix, leading to additional advantageous changes in cellular physiology. These stages of biofilm development are regulated by both environmental and internal signals, with multiple inputs allowing for fine-tuned control (1).

In many bacteria, internal signaling relies on cyclic dinucleotides, such as bis(3′-5′)-cyclic dimeric GMP (c-di-GMP), a small widespread second messenger that regulates numerous bacterial behaviors, including biofilm formation, motility, and virulence (3, 4). c-di-GMP is synthesized by diguanylate cyclases (DGCs), which contain a GGDEF domain, and is degraded by phosphodiesterases (PDEs), containing either EAL or HD-GYP domains (5–7). Although the number of DGCs and PDEs encoded within bacterial genomes can differ greatly, many organisms contain dozens of such genes, highlighting the importance of c-di-GMP as an intracellular signal (3).

c-di-GMP was first discovered as an activator of bacterial cellulose synthase (8). Since then, c-di-GMP has been implicated in activation of numerous polysaccharides in a variety of species at multiple levels of control. For example, in Vibrio cholerae, c-di-GMP activates the *Vibrio* polysaccharide (*vps*) locus through binding and increasing activity of transcriptional regulators VpsR and VpsT (9–12). Activation of polysaccharide production by c-di-GMP is often linked to environmental signals and signal transduction, and sensory domains are commonly associated with DGCs and PDEs (3). This variety of sensory domains is matched by the diversity of known signals that impact c-di-GMP. For example, in V. cholerae alone, known signals include temperature, bile acids, polyamines, and ferrous iron (9, 13–16).

Calcium is one environmental signal linked to c-di-GMP and known to impact bacterial biofilm formation. In marine *Vibrio* spp., the level of calcium present in seawater (10 mM [17]) exerts both negative (V. cholerae [18]) and positive (V. vulnificus [19] and V. fischeri [20]) effects on biofilm formation, all through different mechanisms. In V. cholerae, the calcium-sensing two-component system CarRS decreases transcription of *vpsR*, resulting in decreased *vps* transcription and biofilm formation (18). In V. vulnificus, calcium increases c-di-GMP, increasing transcription of the biofilm and rugose polysaccharide (*brp*) locus, dependent on both the IamA pilin and VpsR homolog, BrpR (19, 21, 22). The calcium-binding matrix protein CabA, which is also induced by calcium, contributes to rugosity and matrix structure (23, 24). In V. fischeri, calcium coordinately induces transcription of genes for the synthesis of two polysaccharides known to contribute to biofilm formation, *bcs* (cellulose) and *syp* (symbiosis polysaccharide [SYP]) (20). The mechanism by which calcium increases transcription of polysaccharide loci and induces biofilm in V. fischeri is as yet unknown; the V. fischeri genome lacks homologs of known calcium-sensing systems present in the other *Vibrio* spp. Calcium also inhibits V. fischeri motility, a behavior considered to be the opposite of sessile biofilm formation (25). While motility control has been associated with changes in c-di-GMP levels, the mechanism by which calcium inhibits motility also is unknown (25, 26). Understanding how V. fischeri biofilm formation and motility are controlled in response to environmental signals, specifically calcium, is important, as both phenotypes represent key behaviors that are required for symbiotic colonization by V. fischeri of its host organism, the Hawaiian bobtail squid, Euprymna scolopes (27–29).

In this study, we investigated the link between calcium and c-di-GMP in V. fischeri, determining that calcium increases c-di-GMP through the activation of DGC CasA. Activated CasA, in turn, inhibits motility and drives cellulose-dependent biofilm formation. Additionally, investigation of the V. cholerae homolog CdgK suggested that CdgK may be inhibited by calcium. Collectively, these results identify CasA as an important regulator and illustrate how integration of calcium and c-di-GMP signals affect bacterial behaviors.

## RESULTS

### Calcium increases c-di-GMP in V. fischeri.

**FIG 1:**
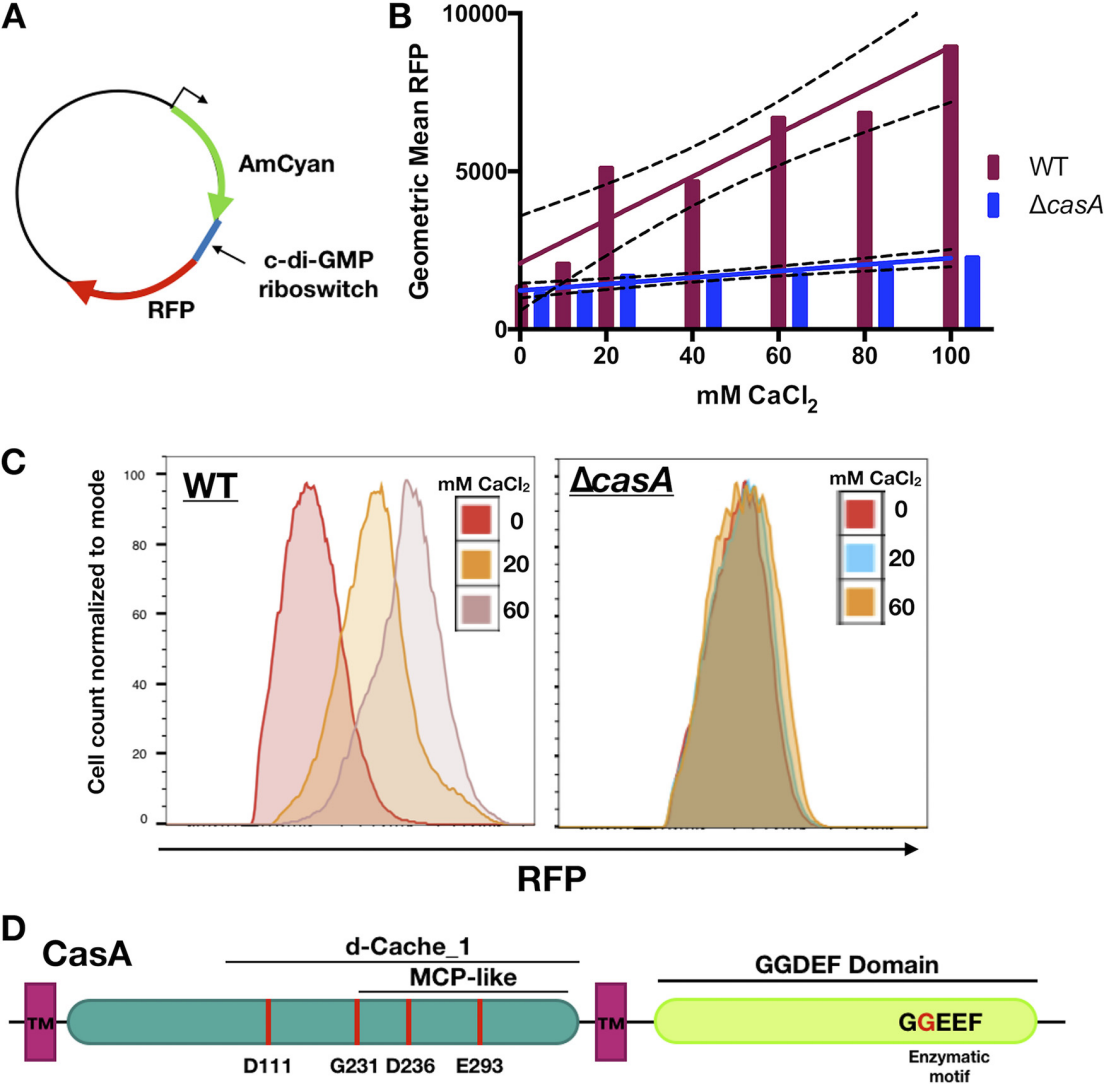
CasA increases intracellular c-di-GMP in response to calcium. (A) Schematic of pFY4535 c-di-GMP biosensor plasmid, which contains a constitutive AmCyan cassette and a c-di-GMP binding riboswitch that controls Turbo-RFP expression. (B) Geometric mean of RFP, the area under the curve as determined via flow cytometry for ES114 (WT) and Δ*casA* (KV9179) containing the pFY4535 biosensor plasmid. Data points represent three biological replicates at increasing concentrations of CaCl_2_ as indicated. Solid lines indicate linear regression fitted slope lines, and dashed lines indicated the confidence interval. Linear regression analysis determined the slopes are significantly different from each other (*P = *0.0001). (C) Representative histograms showing AmCyan^+^ RFP^+^ live cells, with cell count normalized to mode. The *x* axis shows increasing RFP. The left histogram represents ES114 (WT), and the right represents the Δ*casA* mutant (KV9179), both strains containing plasmid pFY4535. (D) Schematic of general CasA protein structure. Two transmembrane regions flank an N-terminal sensory domain (teal) with predicted dCache-1 and MCP-like domains. The C-terminal domain consists of a GGDEF DGC domain containing an enzymatic GGEEF motif. Red lines and letters indicate substituted residues. All locations are approximate and not to scale.

In V. vulnificus, calcium induces biofilm formation through an increase in c-di-GMP (19). Because calcium also induces biofilm formation by V. fischeri (20), we hypothesized that calcium could cause an increase in c-di-GMP. To investigate this possibility, we utilized an intracellular c-di-GMP biosensor in which red fluorescent protein (RFP) production is controlled by a c-di-GMP-dependent riboswitch (30) (Fig. 1A). Calcium supplementation significantly increased RFP levels in biosensor-containing wild-type (WT) strain ES114 (Fig. 1B and C). This increase was dose dependent (Fig. 1B and C), indicating that increasing calcium causes an increase in intracellular c-di-GMP.

The V. fischeri genome contains 50 genes that encode proteins with GGDEF, EAL, or HD-GYP domains, predicted to synthesize, degrade, and/or bind c-di-GMP (31–33). To determine whether any of these genes was responsible for the calcium-dependent increase in c-di-GMP, we performed a preliminary screen evaluating biosensor activity of strains carrying single deletions of each gene. While several mutants exhibited altered responses to calcium, we report here our findings for one gene, the putative diguanylate cyclase gene *VF_1639* (Fig. 1D), which we termed *casA*, for calcium-sensing protein A. In contrast to the WT, a Δ*casA* mutant exhibited minimal changes in RFP levels up to 100 mM CaCl_2_ (Fig. 1B and C), suggesting that CasA is critical for calcium-dependent increases in c-di-GMP.

### CasA inhibits motility in the presence of calcium.

**FIG 2:**
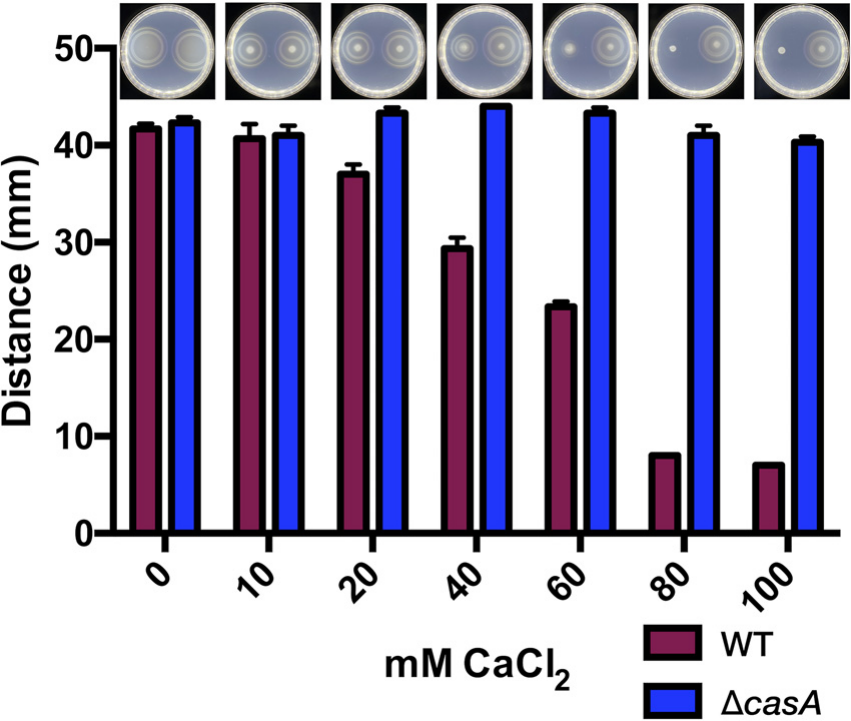
CasA inhibits motility in response to calcium. Migration of WT (ES114) and the Δ*casA* mutant (KV9179) on TBS-Mg^2+^ soft-agar motility plates containing increasing amounts of calcium as indicated is shown. Migration was measured as the diameter of each spot at a 4-h endpoint. Representative images (top) of each plate have WT on the left and the Δ*casA* mutant on the right. The assay was performed at least three independent times.

To explore whether loss of the calcium-dependent increase in c-di-GMP in the Δ*casA* mutant translated into c-di-GMP-related phenotypes, we evaluated motility, a phenotype known to be influenced by calcium levels (25). In unsupplemented motility agar, the Δ*casA* mutant phenocopied WT migration over time (Fig. 2). In the presence of calcium, WT migration decreased in a dose-dependent manner, while migration of the Δ*casA* mutant remained constant and unaffected by concentrations up to 100 mM (Fig. 2). As migration depends on both growth and motility, we evaluated growth of the two strains to determine if differences in migration could be attributed to growth defects or advantages. In the presence of either 40 mM or 100 mM calcium, cells entered stationary phase sooner, but the WT and Δ*casA* strains were equally affected (see Fig. S1 in the supplemental material), indicating that any differences in migration are likely due to motility rather than growth. These data suggest that V. fischeri senses calcium and, in a manner that requires CasA, alters its migration accordingly.

10.1128/mBio.02573-21.2 FIG S1 Growth of V. fischeri strains in high calcium. WT (ES114) or the *casA* mutant strain (KV9179) were grown in TBS without (−) CaCl_2_ or with (+) 40 mM (left) or 100 mM (right) CaCl_2_. Download FIG S1, PDF file, 0.04 MB. Copyright © 2021 Tischler et al. 2021 Tischler et al. https://creativecommons.org/licenses/by/4.0/ This content is distributed under the terms of the Creative Commons Attribution 4.0 International license.

### Calcium enters V. fischeri cells independent of CasA.

**FIG 3:**
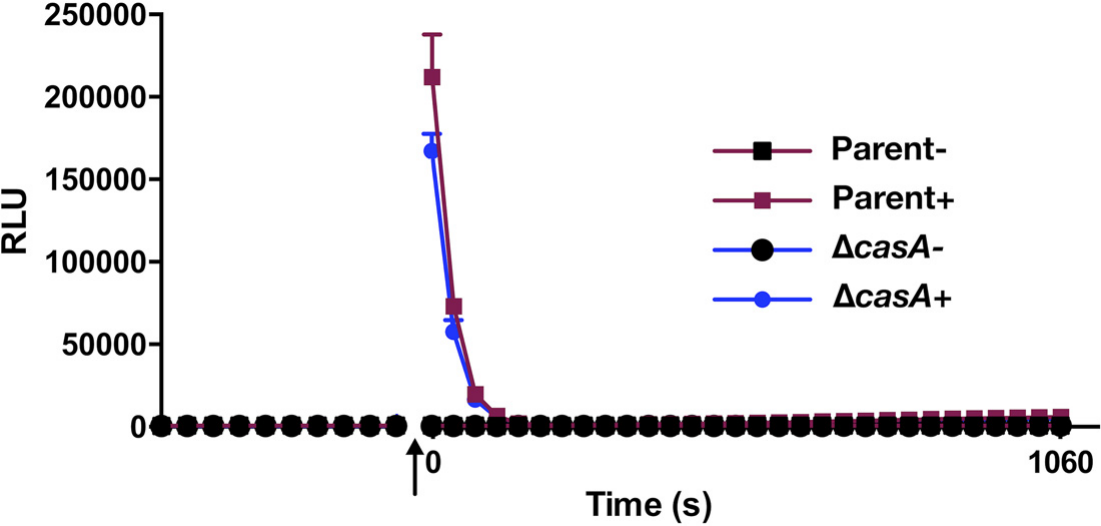
Calcium uptake into bacterial cells occurs independent of CasA. Relative light units (RLU) of the Δ*luxCDABEG* (parent; EVS102) and Δ*luxCDABEG* Δ*casA* (Δ*casA*; KV9817) strains containing apoaequorin-expressing plasmid pAT103 are shown. Coelenterazine was added prior to baseline RLU (left). The arrow indicates that 40 mM calcium was added to “+” wells; “-” indicates no addition. Measurements were taken every few seconds until the endpoint. The graph is representative of at least three independent experiments.

V. fischeri exhibits dose-dependent calcium phenotypes that are lost in a Δ*casA* mutant (Fig. 1 and 2), suggesting the possibility that a Δ*casA* mutant could be defective in calcium uptake. To evaluate if calcium enters cells, and if this uptake depends on CasA function, we expressed aequorin, a cytoplasmic photoprotein that emits light dependent on calcium binding (34, 35), in both the parent (Δ*lux*) and Δ*casA* (Δ*lux*) mutant strains. Upon exposure to 40 mM calcium, the parent strain exhibited a spike in light production, indicating that changes in exogenous calcium are reflected intracellularly (Fig. 3). The Δ*casA* strain behaved similarly to the parent strain, albeit displaying a minor decrease in activity. Thus, the inability of a *casA* mutant to respond to calcium cannot be attributed to an entry defect. Additional work will be necessary to understand the dynamics of calcium uptake; in this study, we focused on elucidating the role of CasA in the signaling network that leads to control of motility and biofilm formation.

### CasA contributes to calcium-dependent biofilm formation.

**FIG 4:**
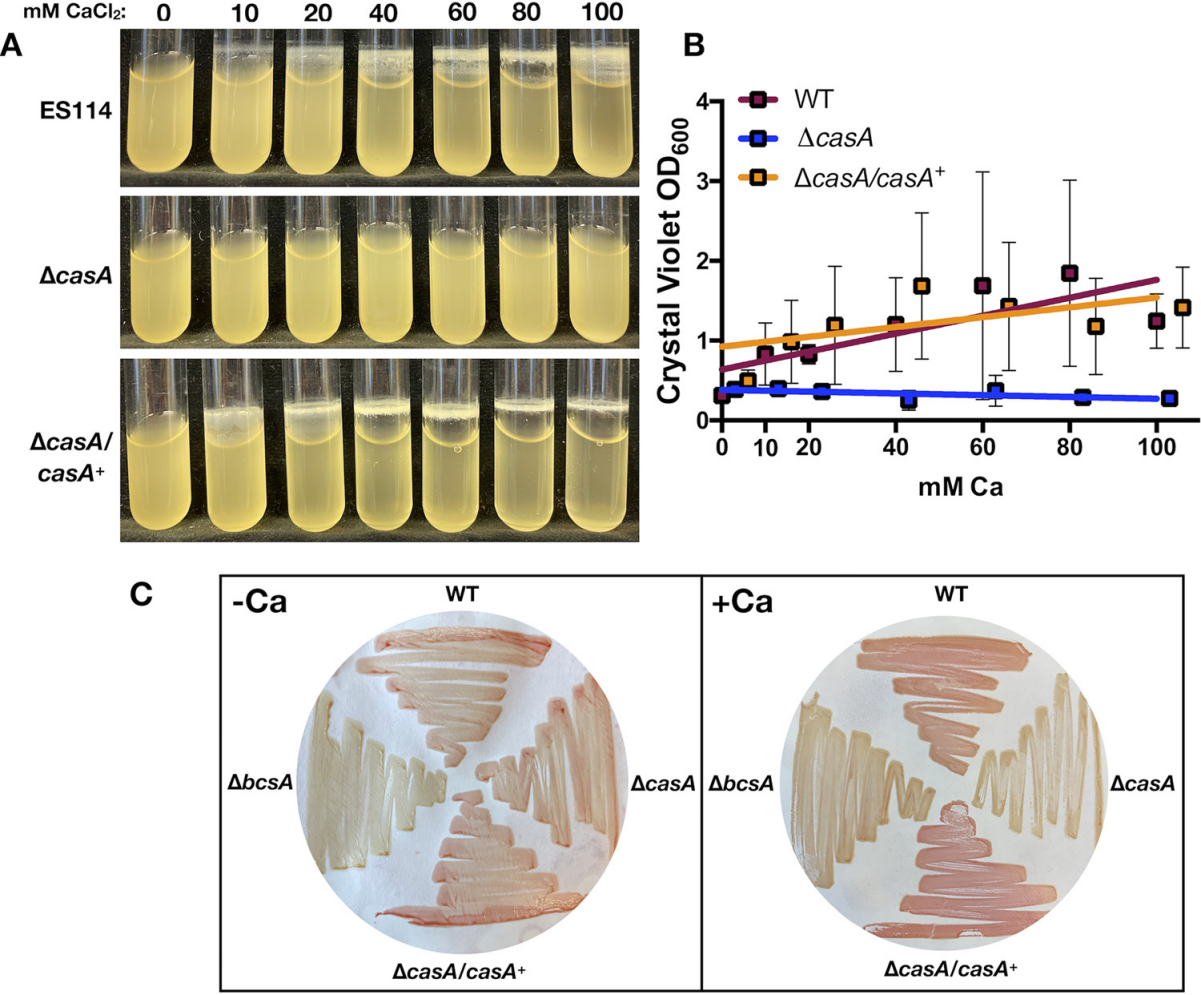
CasA induces cellulose-dependent biofilms in response to calcium. (A) Shaking biofilm assay with increasing amounts of exogenous calcium added to cultures. Strains are ES114 (WT), Δ*casA* (KV9179), and Δ*casA*/*casA^+^* (KV9821). (B) Quantification of crystal violet staining of biofilms like those show in panel A. Symbols represent the mean of each sample and error bars show the standard deviation. Solid lines indicate linear regression analysis, where the lines that represent the WT and the Δ*casA*/*casA^+^* strain are not significantly different from each other but are significantly different from the Δ*casA* mutant (*P = *0.02). (C) Congo red dye bound to bacteria grown on LBS plates without (left) and with (right) added 40 mM calcium. Clockwise from the top, the strains are the WT (ES114) and the Δ*casA* (KV9179), Δ*casA*/*casA^+^* (KV9821), and Δ*bcsA* (KV7894) mutants.

V. fischeri biofilms are induced by calcium (20), but it is unknown if these biofilms depend on CasA. We thus assessed biofilm formation by the WT, Δ*casA*, and complemented Δ*casA* strains grown with increasing amounts of calcium using a shaking liquid biofilm assay. In the presence of 10 mM CaCl_2_, as previously seen (20), the WT formed a small cellulose-associated surface-adherent ring, which became more robust with increasing calcium concentrations (Fig. 4A). Conversely, the Δ*casA* mutant was severely attenuated for ring formation, regardless of calcium levels. Complementation restored the Δ*casA* mutant to a WT phenotype (Fig. 4A). Quantification using crystal violet staining supported the conclusion that the Δ*casA* mutant produced significantly less adherent biomass than both the WT and complemented strains (Fig. 4B and Fig. S2A).

10.1128/mBio.02573-21.3 FIG S2 Evaluation of cellulose production by crystal violet and Congo red staining. (A) Crystal violet staining of V. fischeri strains. Tubes shown in Fig. 4A in the main text were stained with crystal violet, destained, and photographed. WT (ES114), Δ*casA* (KV9179), and Δ*casA*/*casA*^+^ (KV9821) strains were grown in the presence of exogenous CaCl_2_ as indicated at the top. (B to E) Quantification of Congo red dye binding to bacteria via ImageJ. The values for the WT, Δ*casA*, and Δ*casA*/*casA^+^* strains are the same throughout all panels. (B) Strains corresponding to Fig. 4 were normalized, spotted, and quantified. The Δ*bcsA* strain has significantly less binding at 0 mM calcium (*P = *0.0001). At 40 mM calcium, the Δ*bcsA* and Δ*casA* strains are not significantly different, while the Δ*casA* strain has significantly less binding than the WT (*P = *0.0001). (C) Strains corresponding to Fig. 6 were normalized, spotted, and quantified. With 0 mM calcium, there is no significance difference in Congo red binding between strains. With 40 mM calcium, the Δ*casA* and Δ*casA*/*casA-*G410A strains have the same levels of binding, which is significantly less than for the WT (*P = *0.0001). (D) Strains corresponding to Fig. 7 were normalized, spotted, and quantified. With 0 mM calcium, both the Δ*casA*/*casA-*D111A and Δ*casA*/*casA-*D236A strains have significantly more Congo red binding than the WT (*P = *0.0053, 0.0003). (E) Strains corresponding to Fig. 9 were normalized, spotted, and quantified. With 0 mM calcium, Δ*casA*/*cdgK* binds more Congo red dye than the WT, Δ*casA*, or Δ*casA*/*casA^+^* strain (*P* ∼ 0.0002). When 40 mM calcium is added, Δ*casA*/*cdgK* binds the same amount of Congo red dye as its Δ*casA* parent and significantly less than the WT (*P = *0.0038). Download FIG S2, PDF file, 0.4 MB. Copyright © 2021 Tischler et al. 2021 Tischler et al. https://creativecommons.org/licenses/by/4.0/ This content is distributed under the terms of the Creative Commons Attribution 4.0 International license.

Because loss of CasA disrupted cellulose-dependent ring formation, we evaluated the same strains using Congo red, which can bind cellulose. On unsupplemented medium, the strains bound similar amounts of dye, resulting in streaks the same shade of red, while a cellulose mutant (Δ*bcsA*) failed to bind Congo red, resulting in yellow streaks (Fig. 4C, left). On plates with calcium, however, the Δ*casA* mutant produced yellow streaks, phenocopying the Δ*bcsA* mutant, while the WT and complemented Δ*casA* strains streaks were red (Fig. 4C, right). Quantification using ImageJ supported these visual differences (36) (Fig. S2B). Thus, *casA* is required for calcium-induced cellulose production. Additionally, while polysaccharide can decrease motility through steric hindrance (37), the Δ*bcsA* mutant migrated the same as WT, regardless of calcium (Fig. S3), suggesting that cellulose is not responsible for the calcium-mediated motility inhibition.

10.1128/mBio.02573-21.4 FIG S3 Motility of *bcsA* and *vpsR* deletion strains in response to calcium. V. fischeri WT (ES114), Δ*bcsA* (KV7894), and Δ*vpsR* (KV9341) strains were spotted onto motility agar with (+) or without (−) 40 mM CaCl_2_ as indicated. Distances are diameters of spots taken at a 4-h endpoint. Download FIG S3, PDF file, 0.04 MB. Copyright © 2021 Tischler et al. 2021 Tischler et al. https://creativecommons.org/licenses/by/4.0/ This content is distributed under the terms of the Creative Commons Attribution 4.0 International license.

### CasA mediates calcium-dependent increase in *bcs* transcription.

**FIG 5:**
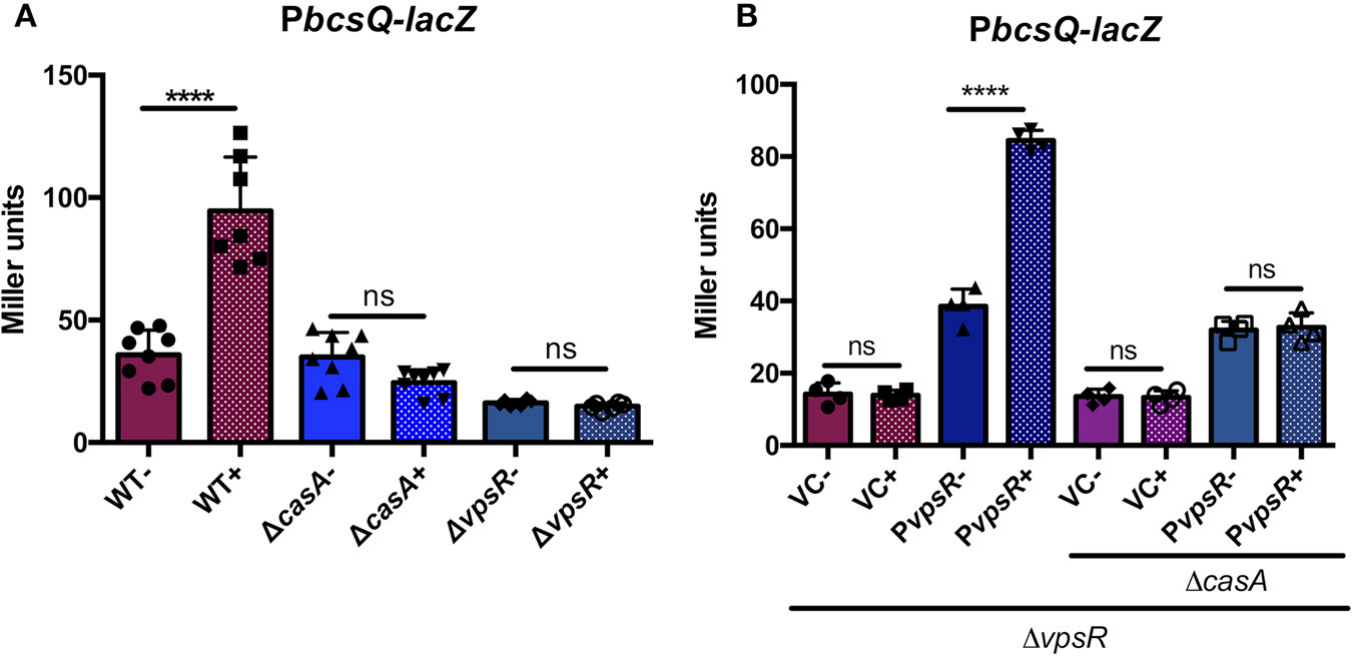
Impact of calcium, CasA, and VpsR on *bcs* transcription. (A) P*bcsQ-lacZ* reporter strains WT (KV8078), Δ*casA* (KV9864), and Δ*vpsR* (KV9865) grown with (+) or without (−) 40 mM added calcium as indicated. Only the WT exhibited a significant increase in *bcsQ* transcription when calcium was added (****, *P = *0.0001). (B) Δ*vpsR* (KV9865) single and Δ*vpsR* Δ*casA* double (KV9868) mutants containing either vector control (VC; pKV69) or *vpsR* overexpression (PvpsR; pCLD42) grown with or without 40 mM added calcium as indicated. Only a Δ*vpsR* mutant strain with pCDL42 exhibited a significant increase in transcription with calcium. Symbols represent individual values, the solid/shaded bars represent the mean of each sample, and error bars show the standard deviations. ns, not significant.

Because calcium induces *bcs* transcription (20), we hypothesized that this effect could depend on CasA. Indeed, calcium induced significant *bcsQ* transcription in the WT but not in the Δ*casA* mutant (Fig. 5A), indicating that CasA is necessary for the calcium-dependent increase in *bcs* transcription. However, as CasA lacks DNA binding domains (Fig. 1D), it most likely acts indirectly.

VpsR was previously linked to the control of cellulose in V. fischeri (38); thus, we hypothesized that it regulates *bcs* transcription. Indeed, *bcsQ* transcription remained low when *vpsR* was deleted, regardless of calcium, suggesting that VpsR is necessary for *bcsQ* transcription (Fig. 5A). Complementation restored *bcsQ* transcription and the calcium-mediated increase in transcription (Fig. 5B). Thus, both *bcsQ* transcription and its induction by calcium require VpsR.

To determine if CasA was epistatic to VpsR, we overexpressed *vpsR* in a Δ*casA* Δ*vpsR* double mutant. *vpsR* overexpression increased *bcsQ* transcription but did not induce a response to calcium, indicating that CasA is needed for the calcium-dependent increase in *bcs* transcription (Fig. 5B). Additionally, *vpsR* transcription was not induced by calcium (Fig. S4A), indicating that control by calcium occurs at a different level. Of note, *vpsR* transcription was unaffected by a *casA* mutation, and it is negatively autoregulated (Fig. S4B to D). Finally, like the Δ*bcsA* mutant, the Δ*vpsR* mutant phenocopied the WT strain for motility in response to calcium (Fig. S3), further suggesting that the CasA-mediated inhibition of motility is independent of cellulose. Overall, these data suggest that VpsR is epistatic to CasA for *bcs* transcription but that the calcium-dependent increase in transcription requires CasA.

10.1128/mBio.02573-21.5 FIG S4 Transcription of the *vpsR* promoter. (A) P*vpsR-lacZ* (KV9573) reporter strain with (+) and without (−) addition of 10 mM CaCl_2_. (B) P*vpsR-lacZ* (KV9573) and Δ*vpsR* P*vpsR-lacZ* (KV9867) strains without any added calcium. WT values are the same in panel A and panel B. (C) P*vpsR*-*lacZ* reporter strain (KV9573) containing either a vector control, pKV69, or a plasmid that overexpresses *vpsR*, pCLD42. (D) P*vpsR-lacZ* reporter strain (KV9573) and Δ*casA* P*vpsR-lacZ* (KV9929). Download FIG S4, PDF file, 0.1 MB. Copyright © 2021 Tischler et al. 2021 Tischler et al. https://creativecommons.org/licenses/by/4.0/ This content is distributed under the terms of the Creative Commons Attribution 4.0 International license.

### CasA is a functional DGC.

CasA contains two domains, an N-terminal periplasmic sensory domain and a C-terminal GGDEF domain (Fig. 1D), suggesting that CasA may both sense a signal(s), such as calcium, and respond accordingly by altering c-di-GMP synthesis. To probe CasA function, we evaluated complementation by hemagglutinin (HA) epitope-tagged point mutant alleles driven by a constitutive promoter and introduced into the chromosome of the Δ*casA* mutant relative to the WT-CasA-complemented strain (Δ*casA*/*casA^+^*). All the resulting variants were produced (Fig. S5A).

**FIG 6:**
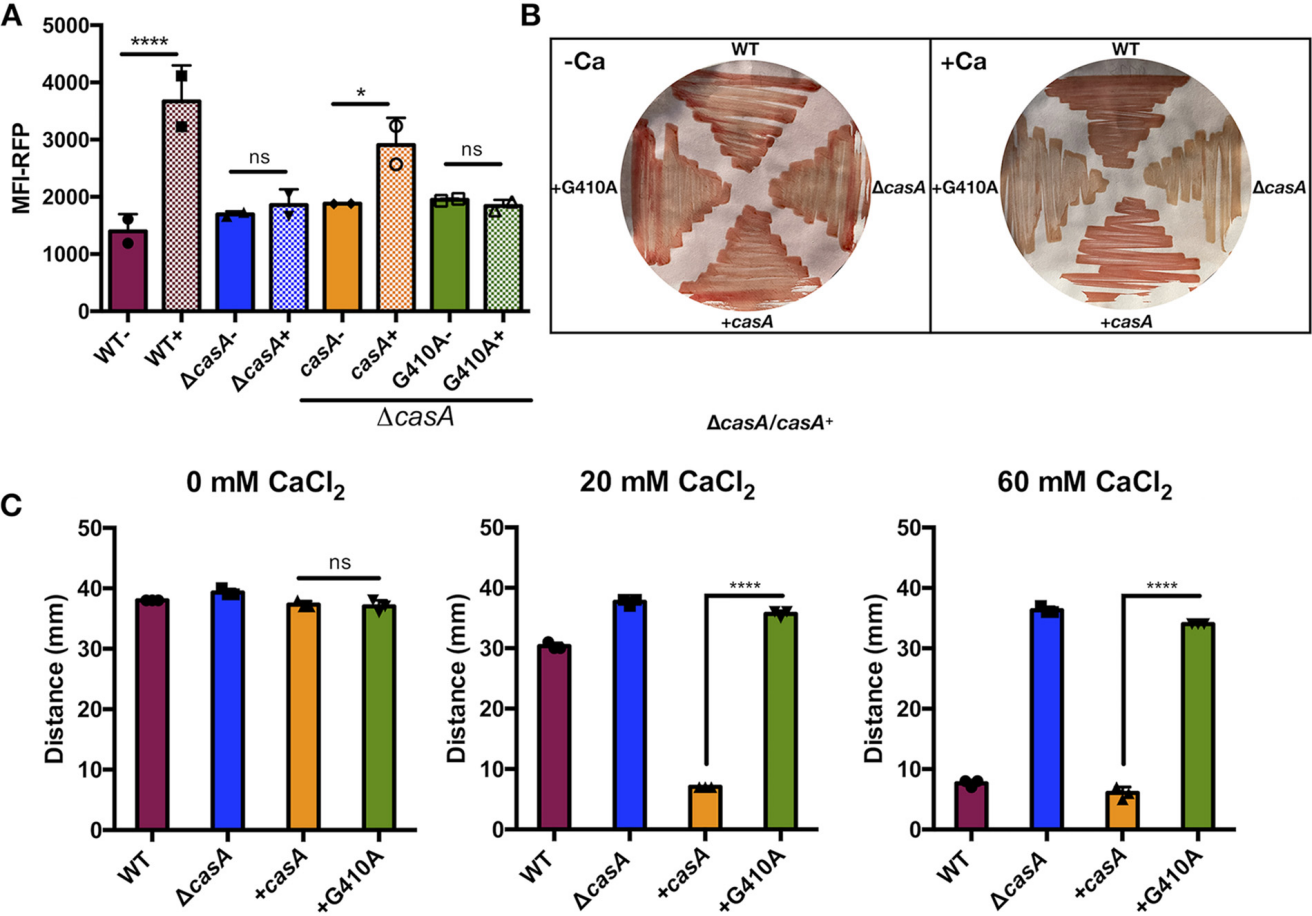
Calcium-dependent phenotypes depend on CasA’s enzymatic GGEEF motif. (A) MFI of RFP in AmCyan^+^ RFP^+^ live cells, with or without 40 mM added calcium. Only the WT and Δ*casA*/*casA^+^* strains increase c-di-GMP in response to calcium (****, *P = *0.0001; *, *P* = 0.01) (B) Congo red dye bound to bacteria grown on LBS plates without (left) or with (right) 40 mM calcium. (C) Migration on motility agar containing 0, 20, or 60 mM added calcium as indicated. When calcium was added, the Δ*casA*/*casA^+^* strain migrated significantly less than the Δ*casA*/*casA-G410A* strain. Strains for all panels are the WT (ES114), Δ*casA* (KV9179), Δ*casA/casA^+^* (*casA*^+^; KV9821), and Δ*casA*/*casA-*G410A (G410A; KV9822) strains. Error bars represent standard deviation. Each assay was independently performed at least three times.

**FIG 7:**
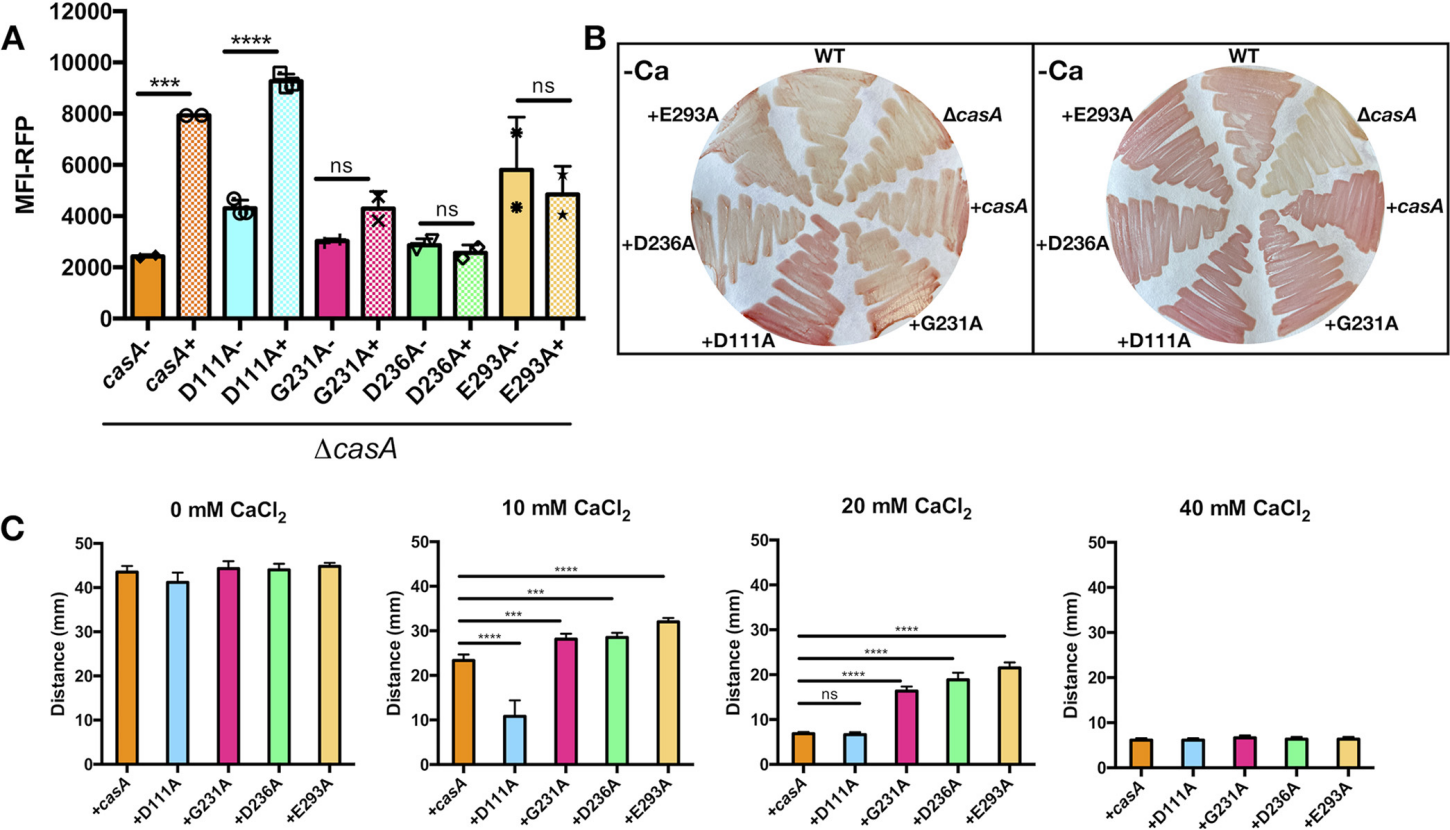
Calcium-dependent phenotypes depend on CasA’s N-terminal sensory domain. (A) MFI of RFP in AmCyan^+^ RFP^+^ live cells, with or without 40 mM added calcium. Only the Δ*casA*/*casA^+^* and Δ*casA*/*casA-D111A* strains increased c-di-GMP in response to calcium (***, *P = *0.002; ****, *P = *0.0001). (B) Congo red dye bound to bacteria grown on LBS plates without (left) or with (right) 40 mM calcium. (C) Migration on motility agar containing 0, 10, 20, or 40 mM calcium as indicated with an endpoint of 5 h. All variants migrated significantly differently than the parent Δ*casA*/*casA^+^* strain at 10 and/or 20 mM calcium addition (***, *P = *0.0003 to 0.0006; ****, *P = *0.0001). Strains for all panels are the Δ*casA*/*casA^+^* (*casA*^+^; KV9821), Δ*casA*/*casA-*D111A (D111A; KV9825), Δ*casA*/*casA-*G231A (G231A; KV9824), Δ*casA*/*casA-*D236A (D236A; KV9826), and Δ*casA*/*casA-*E293A (E293A; KV9827) strains. Error bars represent standard deviation. Each assay was performed independently at least three times.

10.1128/mBio.02573-21.6 FIG S5 Evaluation of CasA and CdgK variants. (A and B) Bacterial strains containing the indicated alleles inserted in the intergenic region between *yeiR* and *glmS* in a Δ*casA* mutant strain were grown in LBS (see methods). Cultures were normalized and lysed samples were run on two gels in tandem; one was stained with Coomassie blue as a loading control (bottom), and the other was transferred and probed with an anti-HA antibody (top). The blot is representative of three replicates. (A) Strains are Δ*casA*/*casA*-HA (KV9821), Δ*casA*/*casA-*G410A-HA (KV9822), Δ*casA*/*casA*-D111A-HA (KV9825), Δ*casA*/*casA*-D236A-HA (KV9826), Δ*casA*/*casA*-E293A-HA (KV9827), Δ*casA*/*cdgK*-HA (KV9828), and Δ*casA*/*casA*-G231A-HA (KV9824) strains. (B) Δ*casA*/*cdgK*-HA (KV9828) grown with the indicated amount of calcium. (C and D) Quantification of the median of the RFP signal corresponding to Fig. 6A (C) and Fig. 7A (D) (**, *P = *0.002; ****, *P = *0.0001). Download FIG S5, PDF file, 0.2 MB. Copyright © 2021 Tischler et al. 2021 Tischler et al. https://creativecommons.org/licenses/by/4.0/ This content is distributed under the terms of the Creative Commons Attribution 4.0 International license.

First, DGC activity was evaluated by generating a G410A substitution in the second glycine of the functional GGEEF motif. c-di-GMP biosensor experiments revealed that the CasA-G410A-expressing strain phenocopied its Δ*casA* parent, failing to increase c-di-GMP levels in response to calcium (Fig. 6A and Fig. S5C). Furthermore, the CasA-G410A variant failed to complement the Congo red and motility phenotypes (Fig. 6B and C and Fig. S2C). Together, these data suggest that c-di-GMP production is necessary for the calcium-dependent biofilm and motility phenotypes controlled by CasA.

### N-terminal sensory domain controls CasA function.

The N-terminal domain of CasA contains dCache-1 (calcium and chemotaxis) and MCP-like (methyl-accepting chemotaxis protein) sensory domains, suggesting a sensory function (Fig. 1D). However, CasA lacks conserved motifs typical of such sensory domains, such as N, F, G1, G2, and G3 boxes (39, 40). Thus, to identify residues potentially important for function, we aligned CasA with homologous proteins from closely related *Vibrio* spp., via BLAST (41), and chose several highly conserved residues to mutate, with a focus on aspartates and glutamates because calcium is known to bind such residues: D111, G231, D236, and E293 (Fig. S6A) (42). Complementation experiments revealed two classes of phenotypes, described below.

10.1128/mBio.02573-21.7 FIG S6 Alignment of homologous proteins. (A) V. fischeri CasA aligned with similar proteins from a variety of *Vibrio* spp., as identified by BLAST. The V. fischeri and V. cholerae proteins are CasA and CdgK, respectively. The residues that were mutated in CasA are highlighted in purple. (B) Alignment between V. fischeri CasA and V. cholerae CdgK. CdgK and CasA are reciprocal best hit when queried against the opposite genome in BLAST. The enzymatic GGEEF motif is highlighted in blue. Asterisks indicate identical residues, colons indicate conserved residues, and periods indicate semiconserved residues. Download FIG S6, PDF file, 0.05 MB. Copyright © 2021 Tischler et al. 2021 Tischler et al. https://creativecommons.org/licenses/by/4.0/ This content is distributed under the terms of the Creative Commons Attribution 4.0 International license.

First, one variant, CasA-D111A, exhibited increased activity while maintaining a response to calcium. Specifically, in the absence of calcium, this strain produced substantially higher levels of c-di-GMP (Fig. 7A, light blue bars, and Fig. S5D) and increased red color on Congo red (Fig. 7B and Fig. S2D). In the presence of 10 mM CaCl_2_, this strain exhibited decreased migration compared to that of the Δ*casA*/*casA^+^* strain (Fig. 7C), indicating a productive response to calcium.

Second, three of the substitutions (G231A, D236A, and E293A) diminished the apparent calcium responsiveness of CasA. All three variants lost the ability to increase c-di-GMP in response to calcium and without calcium maintained similar (G231A and D236A) or slightly higher (E293A) c-di-GMP levels than the Δ*casA*/*casA^+^* strain (Fig. 7A and Fig. S5D). However, these variants phenocopied the Δ*casA*/*casA^+^* strain on Congo red agar containing calcium (Fig. 7B and Fig. S2D), indicating that they must retain some function. In support of this conclusion, they also exhibited intermediate motility phenotypes, with increased migration compared to that of the Δ*casA*/*casA^+^* strain but diminished migration relative to the Δ*casA* mutant at either 10 or 20 mM CaCl_2_ (Fig. 7C). Combining the D236A and E293A substitutions resulted in a strain with slightly increased migration compared to that of each single mutant strain (Fig. S7), suggesting that this combination of substitutions decreases CasA activity but is insufficient to render CasA completely inactive. Overall, these experiments reveal four sensory domain residues that impact CasA function and calcium-dependent phenotypes, suggesting that this domain may be responsible for sensing calcium as a signal.

10.1128/mBio.02573-21.8 FIG S7 Calcium-dependent inhibition of motility by CasA depends on putative N-terminal sensory domain. (A) Δ*casA* (KV9179), Δ*casA*/*casA^+^* (KV9821), Δ*casA*/*casA*-D236A (KV9826), Δ*casA*/*casA*-E293A (KV9827), Δ*casA*/*casA*-D236A-*E293A* (KV9807), and WT (ES114) strains (left to right, respectively) were spotted onto motility plates containing 0 or 20 mM Ca. (B) Zones of migration for the three N-terminal sensory domain mutants were quantified at 4 h. Error bars represent 2 standard deviations. Download FIG S7, PDF file, 0.1 MB. Copyright © 2021 Tischler et al. 2021 Tischler et al. https://creativecommons.org/licenses/by/4.0/ This content is distributed under the terms of the Creative Commons Attribution 4.0 International license.

### CasA is sufficient for the response to calcium.

**FIG 8:**
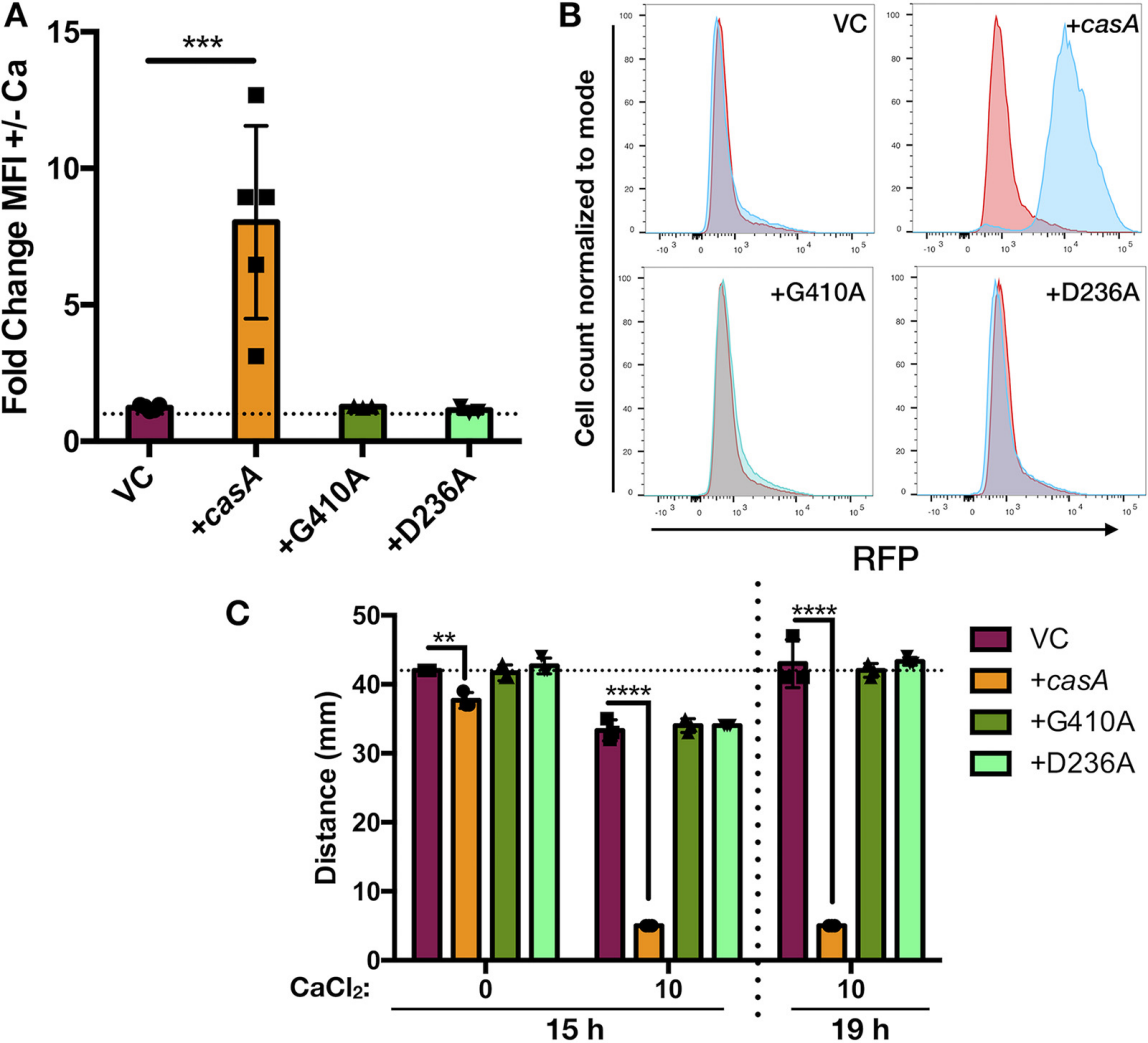
Calcium activates *casA* in E. coli. (A) Fold change of MFI-RFP in response to 10 mM calcium in AmCyan^+^ RFP^+^ live cells. Overexpression of *casA* alone resulted in a significant increase in RFP in response to calcium (***, *P = *0.0006). (B) Representative histograms of the RFP-MFI from panel A with cell count normalized to mode on the *y* axis and increasing RFP on the *x* axis. Red indicates no added calcium, and blue indicates 10 mM calcium. (C) Migration on motility agar with or without 10 mM calcium. The *y* axis represents migration distance, and the *x* axis represents added calcium. Bacteria migrate slower in the presence of calcium, independent of growth (Fig. S8B), so endpoints were chosen when the vector control (VC) reached approximately 40 mm in diameter, which was at 15 h and 19 h for 0 mM and 10 mM calcium, respectively. The horizontal dotted line represents the diameter of the VC with 0 mM calcium to facilitate a comparison of strain migration. Overexpression of *casA* resulted in significantly less migration distance relative to that of the vector control strain (**, *P = *0.003; ****, *P = *0.0001). Neither *casA-G410A* nor *casA-D236A* migrated significantly differently than the vector control. Strains shown contain one of the indicated plasmids: pKV69 (VC), pAT100 (*casA*), pAT101 (*casA-G410A*), pAT102 (*casA-D236A*) in E. coli strains GT115 (A and B), or AJW678 (C). Error bars represent standard deviation. Each assay was performed independently at least three times.

While CasA is responsible for several calcium-dependent phenotypes, it was unclear whether CasA directly senses calcium as a signal or if it requires a partner(s) to sense and respond to calcium. To explore the sufficiency of CasA in response to calcium, we used Escherichia coli as a heterologous system with a high-copy-number plasmid that expressed *casA*. Low calcium levels exerted minimal effects on c-di-GMP levels in E. coli under our conditions (Fig. 8A and B). However, when *casA* was overexpressed, addition of 10 mM calcium resulted in a significant increase in c-di-GMP (Fig. 8A and B). Furthermore, *casA*-overexpressing E. coli failed to migrate in soft agar when calcium was added (Fig. 8C and Fig. S8A). The vector control strain did migrate in the presence of calcium, albeit slower than in its absence, ultimately reaching the same diameter as in the absence of calcium, while the *casA*-overexpressing strain did not progress. The calcium-induced delay in migration was not due to a growth defect (Fig. S8B). These data suggest that CasA alone is sufficient to sense calcium and inhibit motility, presumably by producing c-di-GMP.

10.1128/mBio.02573-21.9 FIG S8 CasA responds to calcium by inhibiting migration in motile E. coli. Motility of motile E. coli strain AJW678 with plasmids pKV69 (VC), pAT100 (*casA^+^*), pAT101 (*casA-*G410A), and pAT102 (*casA-*D236A) is shown. Strains were spotted onto soft-agar TB motility plates that contained either 0 or 10 mM Ca. Motility phenotypes were assessed at 15 h and 19 h by comparing the zones of migration for each strain. (B) Growth of E. coli with 10 mM calcium. Shown are results for strain AJW678 expressing either vector control (VC; pKV69) or *casA* expression plasmid (*casA*; pAT100), during growth in TB either without (−) or with (+) 10 mM CaCl_2_. Download FIG S8, PDF file, 0.08 MB. Copyright © 2021 Tischler et al. 2021 Tischler et al. https://creativecommons.org/licenses/by/4.0/ This content is distributed under the terms of the Creative Commons Attribution 4.0 International license.

To determine if this response (i) was dependent on the ability of CasA to make c-di-GMP and (ii) required a functional sensory domain, we evaluated the G410A enzymatic domain and D236A sensory domain variants. E. coli that expressed either variant phenocopied the vector control strain, with no calcium-dependent change in c-di-GMP levels or migration (Fig. 8 and Fig. S8A). These data suggest that both the periplasmic sensory domain and DGC enzymatic domain are required for CasA to sense and respond to calcium. Thus, CasA appears to be a novel calcium sensor that controls cellulose-dependent biofilm formation via a calcium-mediated induction of c-di-GMP.

### CdgK and CasA respond differently to calcium.

**FIG 9:**
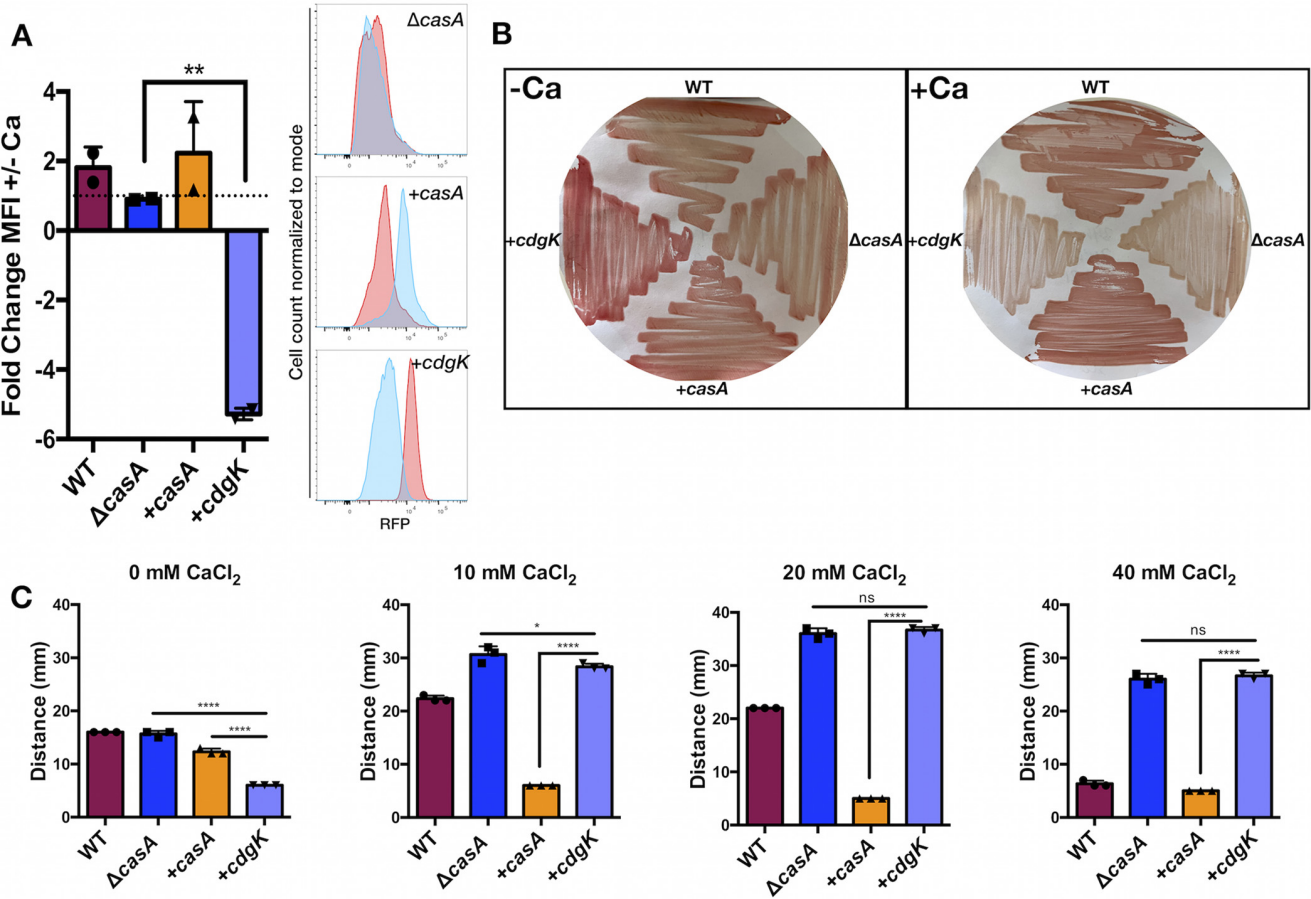
V. cholerae CdgK calcium-dependent phenotypes. (A) Fold change of RFP-MFI (left) in response to calcium and representative histograms (right) as determined by flow cytometric analysis of AmCyan^+^ RFP^+^ live cells containing pFY4535. The Δ*casA*/*cdgK^+^* strain is significantly different from its parent Δ*casA* strain (**, *P = *0.0053). (B) Congo red dye bound to bacteria grown on LBS plates without (left) or with (right) 40 mM calcium. (C) Migration on motility agar containing 0, 10, 20, or 40 mM added calcium as indicated. The Δ*casA*/*cdgK^+^* strain is significantly different from both its parent Δ*casA* strain and the Δ*casA*/*casA^+^* strain in the absence of calcium (****, *P = *0.0001). As calcium increased, Δ*casA*/*cdgK^+^* migrated more similarly to the Δ*casA* strain (*, *P = *0.0436 at 10 mM; not significant at 20 or 40 mM) but continued to migrate significantly differently from the Δ*casA*/*casA^+^* strain under all calcium conditions (****, *P = *0.0001). Strains for all panels are the WT (ES114), Δ*casA* (KV9179), Δ*casA*/*casA^+^* (KV9821), and Δ*casA*/*cdgK^+^* (KV9828) strains. Error bars represent standard deviation. Each assay was performed independently at least three times.

V. fischeri CasA exhibits significant homology to V. cholerae CdgK; when the CasA sequence was used to probe the V. cholerae genome by BLAST, CdgK (VC1104) was the top hit, and vice versa for CdgK and the V. fischeri genome (Fig. S6B). Additionally, the genes for these two proteins are immediately adjacent to a gene (encoding tRNA-dihydrouridine synthase) that is conserved in both genomes, suggesting that they could be orthologs. CdgK is one of a set of DGCs that work upstream of VpsR to activate *vps* transcription (13, 43). These similarities prompted us to explore if *cdgK* could complement the Δ*casA* mutant by expressing *cdgK* in the chromosome of a Δ*casA* mutant. This strain produced CdgK in both the presence and absence of calcium (Fig. S5B). However, it exhibited significantly decreased c-di-GMP levels in response to calcium (Fig. 9A). This suggested that CdgK is an active DGC when expressed in V. fischeri and that this activity may be negatively modulated by exogenous calcium. Consistent with these findings, the *cdgK*-expressing strain showed increased Congo red binding relative to controls in the absence of calcium (Fig. 9B), but with calcium supplementation, it failed to bind Congo red, phenocopying the Δ*casA* parent (Fig. 9B and Fig. S2E).

Despite the impact on c-di-GMP levels and Congo red binding, *cdgK* expression exerted no effect on motility, with or without calcium (Fig. S9). However, when we omitted the motility-promoting magnesium supplement (25), we found that it had masked the impact of *cdgK* on migration. Whereas the migration patterns of the WT, Δ*casA*, and *casA*^+^/Δ*casA* strains mimicked those seen on plates containing magnesium (Fig. 6), the *cdgK*-expressing Δ*casA* strain behaved differently (Fig. 9C). The *cdgK*-expressing strain exhibited significantly less migration in the absence of calcium, but its migration matched that of the uncomplemented Δ*casA* parent strain when calcium was added, indicating a lack of CdgK activity under calcium conditions (Fig. 9C). Together, the observed increases in c-di-GMP levels and Congo red binding and decrease in motility of the *cdgK*-expressing strain in the absence of calcium suggest the expression of an active DGC, while the relative lack of observed activity in the presence of calcium suggests that CdgK activity may be inhibited by calcium.

10.1128/mBio.02573-21.10 FIG S9 V. cholerae CdgK motility in TBS-Mg^2+^. Zones of migration for strains of interest grown on TBS-Mg^2+^ motility plates were measured, quantified, and graphed. Measurements were taken 4 h after spotting each strain. Strains included the WT (ES114), Δ*casA* (KV9179), Δ*casA*/*casA^+^* (KV9821), and Δ*casA*/*cdgK*^+^ (KV9828) strains. Error bars represent 2 standard deviations. Download FIG S9, PDF file, 0.04 MB. Copyright © 2021 Tischler et al. 2021 Tischler et al. https://creativecommons.org/licenses/by/4.0/ This content is distributed under the terms of the Creative Commons Attribution 4.0 International license.

## DISCUSSION

This study investigated how calcium changes V. fischeri behavior. We determined that increasing concentrations of calcium resulted in increased cellulose-dependent biofilm formation and decreased motility, corresponding to increasing concentrations of c-di-GMP. CasA, one of 33 putative DGCs encoded by V. fischeri, was solely responsible; the observed effects were lost in the absence of CasA, and heterologous expression of *casA* in E. coli similarly resulted in calcium-dependent phenotypes. For biofilm formation, the calcium-dependent increase in *bcs* transcription required both CasA and VpsR. Finally, the V. cholerae homolog, CdgK, did not complement CasA but instead exhibited an inverse response, with increased activity in the absence of calcium.

c-di-GMP is a widespread bacterial signal that permits many organisms to change their behavior in response to varied internal and external signals. Calcium was one of the first signals identified as an activator of c-di-GMP through inhibition of PDE activity in Komagataeibacter xylinus, even before the discovery of c-di-GMP in 1987 (8, 44, 45). In the intervening decades, the connection between calcium and c-di-GMP has been investigated in a variety of organisms and signaling pathways. For example, in Mycobacterium tuberculosis, calcium alters PDE activity, affecting growth and survival during macrophage infection (46–48). In the c-di-GMP-activated Lap systems of Pseudomonas aeruginosa and Legionella pneumophila, calcium activates the protease LapG, which promotes biofilm dispersal (49, 50). Additionally, similar to what we observed in this study, calcium increases intracellular c-di-GMP levels in V. vulnificus (19). Our work adds to this literature by identifying CasA as a DGC whose activity is induced in response to calcium. Other DGCs and PDEs responsive to calcium in a variety of organisms are likely to be uncovered as the connection between calcium and c-di-GMP continues to be explored.

The architecture of CasA includes a putative N-terminal periplasmic sensory domain—putatively involved in binding calcium—and a well-conserved C-terminal cytoplasmic GGDEF enzymatic domain responsible for c-di-GMP production. Residues in both of these domains are required for function. Protein prediction programs identify both dCache-1 (calcium and chemotaxis) and MCP-like (methyl-accepting chemotaxis protein) domains in the N terminus. Class I MCPs have a periplasmic ligand binding domain (LBD), with another, functional domain in the cytoplasm (51), matching the general structure of CasA. A screen of calcium-binding proteins identified aspartates and glutamates as the most common calcium-coordinating residues (42). Consistent with those known interactions, N-terminal CasA residues D236 and E293 likely contribute to sensing/binding calcium in some way, as mutating these residues resulted in partial loss of the calcium response. Conversely, mutation of D111 allowed for an increase in basal CasA function without disrupting the ability to sense/respond to calcium. Structural analysis of CasA could provide insight into where and how calcium binds to the N-terminal LBD.

CasA responds to the calcium signal by synthesizing c-di-GMP to control cellulose production. A wide variety of bacterial species synthesize cellulose polysaccharide, leading to a diversity of *bcs* operon structures, genes, cellulose products, and regulatory mechanisms. But throughout this heterogeneity, bacterial cellulose remains inextricably linked to c-di-GMP, as all identified BCS complexes contain a BcsA subunit with a PilZ c-di-GMP binding domain (52). E. coli and Salmonella spp. are the best-studied organisms with type II *bcs* operons, the same kind found in V. fischeri. In V. fischeri, regulation starts at the transcriptional level, where *bcs* transcription depends on VpsR and is significantly increased by calcium due to the DGC CasA. Without CasA, calcium has no impact on either the internal levels of c-di-GMP or *bcs* transcription. Conversely, in E. coli and Salmonella spp., *bcs* transcription is thought to be constitutively active and therefore unregulated (53). However, posttranscription regulation of cellulose by c-di-GMP is well established in these organisms, with c-di-GMP binding to and allosterically activating BcsA promoting synthesis and binding to BcsE, activating BcsG for postsynthetic modification (54, 55). While this has not yet been investigated, c-di-GMP almost certainly regulates cellulose synthesis in V. fischeri by binding to and activating the predicted BcsA and BcsE proteins, perhaps due to CasA-relayed calcium and/or additional signals. The PDE BinA is known to impact cellulose polysaccharide and represents another candidate for cellulose regulation at the synthesis or postsynthetic level (56).

Regulation of the *bcs* locus in V. fischeri contains parallels to regulation of other *Vibrio* polysaccharide loci and is often directly inverse to regulation in V. cholerae. First, calcium affects transcription of a major polysaccharide locus in at least three different *Vibrio* spp., activating *bcs* and *brp* transcription in V. fischeri and V. vulnificus, respectively, and downregulating *vps* transcription in V. cholerae (18–20). Calcium acts to increase c-di-GMP in both V. fischeri and V. vulnificus, activating CasA in V. fischeri to increase *bcs* transcription (19). The specific calcium-dependent mechanism activating *brp* transcription is yet unknown but also dependent on c-di-GMP (19). Conversely, in V. cholerae, *vps* transcription is decreased by calcium through downregulation of *vpsR* transcription by the calcium-sensing two-component system CarRS (18). Second, the homologous transcriptional regulators VpsR in both V. fischeri and V. cholerae autoregulate their own transcription, albeit in opposite directions, with V. fischeri VpsR negatively autoregulating and V. cholerae VpsR engaging in positive autoregulation (57). Third, the homologous DGCs CasA and CdgK respond oppositely to calcium, with CasA increasing and CdgK decreasing c-di-GMP levels in the presence of calcium, paralleling the effects on *bcs* and *vps* transcription. In V. cholerae, VpsR activity is increased by c-di-GMP (11, 12). This is likely to be the case for the V. fischeri protein as well; if so, this could account for CasA-mediated induction of *bcs* transcription in response to calcium. Overall, these differences in regulation speak to the evolutionary divergence between species, with the same signal allowing each organism to adapt to its environmental niches without major functional changes.

In summary, this study identifies CasA as a calcium-sensing DGC in V. fischeri, responsible for inducing cellulose-dependent biofilm formation and inhibiting motility in response to calcium. CasA is required for a calcium-dependent increase in *bcs* transcription, connecting c-di-GMP to transcriptional control of a type II *bcs* locus. The V. cholerae homolog, CdgK, and CasA are not equivalent, but the enzymatic activity of both proteins seems to be modulated by calcium, providing insight into how related species can adapt to their specific niches.

## MATERIALS AND METHODS

### Strains and media.

V. fischeri strains, plasmids and primers used in this study are listed in Tables 1 to 3. All strains used in this study were derived from strain ES114, a bacterial isolate from *Euprymna scolopes* (58). Escherichia coli strains TAM1 (Active Motif), TAM1 λ *pir* (Active Motif), DH5α, π3813, and GT115 were used for cloning (59). For routine culturing, V. fischeri strains were grown in LBS (60, 61) and E. coli strains were grown in LB (62), in some cases supplemented with thymidine. For natural transformation of V. fischeri, Tris minimal medium (TMM) (100 mM Tris [pH 7.5], 300 mM NaCl, 0.1% ammonium chloride, 10 mM *N*-acetylglucosamine, 50 mM MgSO_4_, 10 mM KCl, 10 mM CaCl_2_, 0.0058% K_2_HPO_4_, 10 μM ferrous ammonium sulfate) was used. Soft-agar motility medium (tryptone broth [TB] for *E. coli* and tryptone broth salt [TBS] for *V. fischeri*) contained tryptone (1%), NaCl (2% for V. fischeri and 1% for E. coli), agar (0.25%), and MgSO_4_ (35 mM), and CaCl_2_ was added to the desired concentration. Antibiotics were added as appropriate at the following final concentrations: ampicillin (Amp), 100 μg ml^−1^; chloramphenicol (Cm), 1 μg ml^−1^; erythromycin (Em), 2.5 μg ml^−1^ (V. fischeri) or 150 μg ml^−1^ (E. coli); kanamycin (Kn), 100 μg ml^−1^ (V. fischeri) or 50 μg ml^−1^ (E. coli); spectinomycin (Sp), 200 μg ml^−1^; and gentamicin (Gen), 5 μg ml^−1^. For Sp and Gen, LB was used as the medium instead of LBS for outgrowth and plating.

**TABLE 1:** Strains used in this study

Strain	Genotype^*a*^	Construction^*b*^	Reference
AJW678	*thi-1 thr-1*(Am) *leuB6 metF159*(Am) *rpsL136* Δ*lacX74*		75
ES114	WT		58
EVS102	Δ*luxCDABEG*		76
KV7371	IG::P*sypA-lacZ*		77
KV7894	Δ*bcsA*		20
KV8078	Δ*sypQ*::FRT-Cm^r^ attTn*7*::P*bcsQ-lacZ*		20
KV8232	IG (*yeiR-glmS*)::Erm^r^-trunc Trim^r^		68
KV8920	Δ*casA*::FRT-Spec^r^	TT ES114 via intermediate strain generated with SOE using primers 2561 and 2562 (ES114), 2089 and 2090 (pKV521), and 2563 and 2564 (ES114)	This study
KV9179	Δ*casA*::FRT	KV8920 with Spec^r^ cassette removed using pKV496	This study
KV9341	Δ*vpsR*::FRT-Spec^r^	TT ES114 with SOE using primers 2093 and 2094 (ES114), 2089 and 2090 (pKV521), and 2095 and 2096 (ES114)	This study
KV9573	IG::P*vpsR-lacZ*	TT KV7371 with SOE using primers 2185 and 2090 (pKV502), 2932 and 2933 (ES114), and 2822 and 2876 (KV7371); replaces P*sypA*-*lacZ*	This study
KV9807	Δ*casA*::FRT-Spec^r^ IG::P*nrdR-casA*-*D236A*-*E293A*-HA	TT KV9818 with SOE using primers 2290 and 3122 (KV9826) and 3121 and 1487 (KV9827)	This study
KV9817	Δ*luxCDABEG* Δ*casA*::FRT-Spec^r^	TT EVS102 with gKV9821	This study
KV9818	Δ*casA*::FRT-Spec^r^ IG::Erm^r^-trunc Trim^r^	TT KV8232 with gKV8920	This study
KV9820	Δ*casA*::FRT-Spec^r^ IG::P*nrdR*-*casA*-HA	TT KV9818 with SOE using primers 2290 and 2090 (pKV506), 2905 and 3042(ES114), and 2089 and 1487 (pKV505)	This study
KV9821	Δ*casA*::FRT-Spec^r^ IG::P*nrdR*-RBS-*casA-*HA	TT KV9818 with SOE using primers 2290 and 2090 (pKV506) and 3057 and 1487 (KV9820)	This study
KV9822	Δ*casA*::FRT-Spec^r^ IG::P*nrdR*-RBS-*casA-G410A-*HA	TT KV9818 with SOE using primers 2290 and 2911 (KV9821) and 2910 and 1487 (KV9821)	This study
KV9824	Δ*casA*::FRT-Spec^r^ IG::P*nrdR*-RBS-*casA-G231A-*HA	TT KV9818 with SOE using primers 2290 and 3112 (KV9821) and 3111 and 1487 (KV9821)	This study
KV9825	Δ*casA*::FRT-Spec^r^ IG::P*nrdR*-RBS-*casA-D111A-*HA	TT KV9818 with SOE using primers 2290 and 3118 (KV9821) and 3117 and 1487 (KV9821)	This study
KV9826	Δ*casA*::FRT-Spec^r^ IG::P*nrdR*-RBS-*casA-D236A-*HA	TT KV9818 with SOE using primers 2290 and 3120 (KV9821) and 3119 and 1487 (KV9821)	This study
KV9827	Δ*casA*::FRT-Spec^r^ IG::P*nrdR*-RBS-*casA-E293A-*HA	TT KV9818 with SOE using primers 2290 and 3122 (KV9821) and 3121 and 1487 (KV9821)	This study
KV9828	Δ*casA*::FRT-Spec^r^ IG::P*nrdR*-RBS-*cdgK*-HA	TT KV9818 with SOE using primers 2290 and 2090 (pKV506), *cdgK* gblock (IDT), and 2089 and 1487 (pKV505)	This study
KV9864	Δ*sypQ*::FRT-Cm^r^ Δ*casA*::FRT-Spec^r^ attTn*7*::P*bcsQ-lacZ*	TT KV8078 with gKV8920	This study
KV9865	Δ*sypQ*::FRT-Cm^r^ Δ*vpsR*::FRT-Spec^r^ attTn*7*::P*bcsQ-lacZ*	TT KV8078 with gKV9341	This study
KV9866	Δ*casA*::FRT Δ*vpsR*::FRT-Spec^r^	TT KV9179 with gKV9341	This study
KV9867	Δ*vpsR*::FRT-Spec^r^ IG::P*vpsR-lacZ*	TT KV9573 with gKV9341	This study
KV9868	Δ*casA*::FRT Δ*vpsR*::FRT-Spec^r^ attTn*7*::P*bcsQ-lacZ*	Derived from KV9866 using pCMA26 (20)	This study
KV9929	Δ*casA*::FRT-Spec^r^ IG::P*vpsR-lacZ*	TT KV9573 with gKV8920	This study

a Abbreviations: HA, HA epitope tagged; IG, intergenic between *yeiR* and *glmS* (adjacent to the Tn*7* site); FRT, the antibiotic cassette was resolved using Flp recombinase, leaving a single FRT sequence.

b Derivation of strains constructed in this study. TT, TfoX-mediated transformation of a *tfoX*-overexpressing version of the indicated strain with the indicated genomic DNA (gDNA) or with a PCR-SOE product generated using the indicated primers and templates.

**TABLE 2:** Plasmids used in this study

Name	Description	Derivation^*a*^	Reference
pAT100	pJET + FRT-Erm^r^ *casA-*HA	pJET + PCR product with primers 975 and 1487 (KV9821)	This study
pAT101	pJET + FRT-Erm^r^ *casA+G410A-*HA	pJET + PCR product with primers 975 and 1487 (KV9822)	This study
pAT102	pJET + FRT-Erm^r^ *casA+D236A-*HA	pJET + PCR product with primers 975 and 1487 (KV9826)	This study
pAT103	Apoaequorin, Cm^r^	pVSV105 + apoaequorin	This study
pCLD42	pKV69 + *vpsR*		38
pCMA26	P*bcsQ-lacZ* reporter		20
pFY4535	c-di-GMP biosensor; Gen^r^ *hok*/*sok*		30
pJJC4	*tfoX^+^ +* Cm^r^		66
pKV69	Vector; Cm^r^ Tet^r^		78
pKV496	Kan^r^ + *flp^+^*		68
pKV502	pJET + *yeiR*-FRT-Erm^r^		68
pKV505	pJET + HA-*glmS*		68
pKV506	pJET + yeiR-FRT-Erm^r^-P*nrdR*		68
pKV521	pJET + FRT-Spec^r^		68
plostfoX-Kan	*tfoX^+^ +* Kan^r^		64
pVSV105	Vector; Cm^r^		73

a Derivation of plasmids generated in this study.

**TABLE 3:** Primers used in this study

Primer no.	Sequence^*a*^
975	CCTCACCCCAGATGGTTTGGCA
1487	GGTCGTGGGGAGTTTTATCC
2089	CCATACTTAGTGCGGCCGCCTA
2090	CCATGGCCTTCTAGGCCTATCC
2093	ATCACAGCTCTTGAGCATGG
2094	TAGGCGGCCGCACTAAGTATGGTTGAGTACCCATAACACTACCTC
2095	GGATAGGCCTAGAAGGCCATGGAGCTATAGCTAATCGAATCCTTATTG
2096	CTGGCAGTAAACCTTTACCTG
2185	CTTGATTTATACAGCGAAGGAG
2290	AAGAAACCGATACCGTTTACG
2561	GCGCAGCAATTGATTACAGC
2562	taggcggccgcactaagtatggCAACACTAGGAATAGTGTTGG
2563	ggataggcctagaaggccatggGGTAAAAACCGAGTTTACTTTTC
2564	CTAACCATTCATGCAAGAACC
2822	AGGAAACAGCTatgACCATGATTACGGATTCAC
2876	GAAACGCCGAGTTAACGCC
2905	ggataggcctagaaggccatggCACTTCGTGTTAAAGAATTTATAC
2910	CGATTTGGCGCTGAAGAATTTGTTATCTGTATTAATG
2911	CAAATTCTTCAGCGCCAAATCGAGACACAATATC
2932	ggataggcctagaaggccatggCCATCAATGCGTCCACAAGC
2933	catggtcatagctgtttcctCATAACACTACCTCTAAATTCTTATATC
3042	ttatgcataatctggaacatcatatggataTGAAAAGTAAACTCGGTTTTTACC
3057	ggataggcctagaaggccatggAGGAGGATTTATACATGCCGAAATTTAATTTAAAAC
3111	GTATATTCAAAGCTGTATTGGTCATTGATCTTTC
3112	GACCAATACAGCTTTGAATATACCTTTATAG
3117	GGACGTCTTGCTTATAGTATCGCTGGTAAAAAAG
3118	GATACTATAAGCAAGACGTCCTTCTGCAAC
3119	GTATTGGTCATTGCTCTTTCAGTTGAAAAGC
3120	CTGAAAGAGCAATGACCAATACACCTTTG
3121	GATAAAAGAAGCGTTGAAAGGTTTTCTTGTG
3122	CTTTCAACGCTTCTTTTATCGATGTTTTG

a Lowercase letters indicate nonnative or tail sequences.

### Molecular techniques and strain construction.

Mutations in ES114 were generated through TfoX-mediated transformation (63–66). Briefly, ∼500-bp segments upstream and downstream of genes of interest were PCR amplified using high-fidelity KOD polymerase (Novagen, EMD Millipore), and PCR splicing by overlap extension (SOE [67]) was used to fuse segments to an antibiotic cassette as described previously (68). The fused product was amplified and transformed into the recipient V. fischeri strain (typically ES114) carrying a TfoX-overproducing plasmid (plostfoX [64], plostfoX-Kan [64], or pJJC4 [66]), and recombinant cells were selected on media containing the appropriate antibiotic. The allelic replacement was confirmed by PCR with outside primers using Promega *Taq* polymerase (Table 3). After the initial deletion was made, genomic DNA (gDNA) was isolated from the recombinant strains using the Quick-DNA Miniprep plus kit (Zymo Research) and used to introduce the mutation into other desired strain backgrounds. Insertion at the Tn*7* site was performed via tetraparental mating (69) between the V. fischeri recipient and three E. coli strains, carrying the conjugal plasmid pEVS104 (70), the Tn*7* transposase plasmid pUX-BF13 (71), and pCMA26 (20). Insertions were also introduced adjacent to the Tn*7* site at the intergenic (IG) region between *yeiR* and *glmS* as previously described (68). These insertions were made using the PCR amplification and SOE method described above, with genes of interest fused to an upstream Erm^r^ cassette for selection, driven by the constitutive P*ndrR* promoter, and containing an idealized ribosome binding site (RBS). In some cases, the antibiotic resistance cassette was removed from V. fischeri deletion mutants using Flp recombinase, which acts on Flp recombination target (FRT) sequences to delete the intervening sequences, as has previously been shown (72). Overexpression plasmids were constructed by amplifying genes of interest from the IG region of V. fischeri strains, and the resulting PCR product was ligated into the pJET1.2 blunt cloning vector (Thermo Fisher), transformed into chemically competent E. coli DH5α, and selected using Amp. The resulting plasmids were sequenced (Integrated DNA Technologies), purified using the plasmid miniprep kit (Zymo Research), and transformed into chemically competent *E. coli* FT115 containing either the pFY4535 biosensor or AJW678. The apoaequorin plasmid was synthesized by GenScript using sequence from Aequorea victoria for apoaequorin, clone UTAEQ04, and inserted into plasmid pVSV105 (73).

### c-di-GMP biosensor assay.

Relative c-di-GMP levels were assessed in either LBS or LB broth at 24 or 28°C for V. fischeri and E. coli, respectively, and cultures inoculated from single colonies contained added calcium chloride as indicated. The biosensor was not selected using antibiotics, as it contains a toxin/antitoxin system (30), but Amp was used to select for overexpression plasmids in E. coli strains. Samples were diluted 1:1,000 in phosphate-buffered saline (PBS) and assessed via flow cytometry on a LSRFortessa (BD Biosciences). Forward scatter (FSC) and side scatter (SSC) were collected in a log scale with a threshold of 200, and AmCyan and phycoerythrin (PE)-Texas Red channels were used to measure AmCyan and RFP, respectively. Data were analyzed using FlowJo 10, gating first on live cells as determined by FSC and SSC, then AmCyan to confirm singlets, and finally RFP to assess relative c-di-GMP. This resulting population was used to create representative histograms, and the geometric mean fluorescence intensity (MFI) of each curve was used to quantify and compare samples. Data were graphed using GraphPad Prism 6 and analyzed via linear regression or one-way analysis of variance (ANOVA) as indicated.

### Aequorin assay.

The Δ*luxCDABEG* strain (parent) and its Δ*casA* derivative carrying pAT103 were grown overnight at 28°C in LBS with Cm. The overnight cultures were subcultured 1:100, grown until mid-log phase, and collected and centrifuged. Samples were washed two times and resuspended in PBS, and a limiting amount of coelenterazine (Nanolight Technology) was added to a final concentration of 5 μM. The samples were vortexed and incubated in the dark for 1 h and washed in PBS, and the optical density at 600 nm (OD_600_) was measured. Samples were normalized to an OD of 0.4 in a white 96-well plate and incubated in the dark for 10 min. The baseline luminescence of the samples was measured with a delay of 1 s for 5.9 min in a luminometer (Veritas microplate luminometer; Turner Biosystems). Calcium was added to a final concentration of 40 mM, and the luciferase activity was measured with a delay of 1 s for approximately 17 min 40 s. The limiting amount of coelenterazine is responsible for the temporal appearance of light production. This assay was performed at least three separate times.

### Motility assay.

Single colonies were inoculated in either TBS or TB for V. fischeri and E. coli, respectively. Cultures were grown overnight shaking at 28°C, subcultured 1:100 in fresh broth, and incubated with shaking until exponential growth phase. Cultures were normalized to a final OD_600_ of 0.2, and 10-μl aliquots were spotted onto soft-agar motility plates supplemented with the desired concentrations of CaCl_2_. E. coli plasmids were maintained with Amp throughout. Plates were incubated at 28°C, and the diameter of each zone of migration was measured and imaged at 4 h unless indicated otherwise for V. fischeri and 15 h and 19 h for E. coli. Pictures were taken using an iPhone 11 front-facing camera. Data were analyzed using one-way ANOVA in GraphPad Prism 6. Each experiment utilizing this assay was performed at least three independent times.

### Shaking biofilm assay.

To assess calcium-induced biofilm formation under shaking liquid conditions, LBS broth containing between 10 and 100 mM calcium chloride (as indicated) was inoculated with single colonies of V. fischeri strains and grown with shaking overnight at 24°C. For these experiments only, test tubes (13 by 100 mm) were used with a culture volume of 2 ml of LBS broth. For crystal violet staining, 200 μl of a 1% crystal violet solution was added to cultures for 30 min. Tubes were washed with deionized H_2_O and destained with ethanol. OD_600_ was measured using a Synergy H1 microplate reader (BioTek). Pictures were captured via an iPhone 12 minicamera, and data are representative of at least 3 independent experiments. Linear regression analysis was performed in GraphPad Prism 6.

### Congo red assay.

Bacteria were streaked onto LBS plates containing Congo red and Coomassie blue dyes (40 μg ml^−1^ and 15 μg ml^−1^, respectively), and 40 mM calcium as indicated, and grown overnight at 24°C. To better visualize color differences, cells were transferred onto white paper in a replica plating-like approach by briefly smoothing the paper onto the agar plate and then lifting it off (68). The result was photographed with an iPhone 12 minicamera. For quantification, 10-μl aliquots of culture normalized to an OD of 0.2 were spotted and grown as described above. Spots were compared by assessing the gray values via ImageJ (36).

### β-Galactosidase assay.

Strains carrying a *lacZ* reporter fusion to the *bcsQ* or *vpsR* promoter were grown in duplicate at 24°C in LBS containing calcium chloride as indicated. Strains were subcultured into 20 ml of fresh medium in 125-ml baffled flasks, and samples were collected after 4 h of growth. OD_600_ was measured, and cells were resuspended in Z buffer and lysed with chloroform. The β-galactosidase activity of each sample was assayed as described previously (74) and measured using a Synergy H1 microplate reader (BioTek). Assays were performed at least 2 independent times and analyzed via one-way ANOVA in GraphPad Prism 6.

10.1128/mBio.02573-21.1 TEXT S1 Supplemental methods. Download Text S1, PDF file, 0.09 MB. Copyright © 2021 Tischler et al. 2021 Tischler et al. https://creativecommons.org/licenses/by/4.0/ This content is distributed under the terms of the Creative Commons Attribution 4.0 International license.
