# Supplementary figures and images for: Calcium-Responsive Diguanylate Cyclase CasA Drives Cellulose-Dependent Biofilm Formation and Inhibits Motility in Vibrio fischeri
Source: mBio. 2021 Nov 9;12(6):e02573-21. doi: 10.1128/mBio.02573-21 (PMC8576532; doi:10.1128/mBio.02573-21)

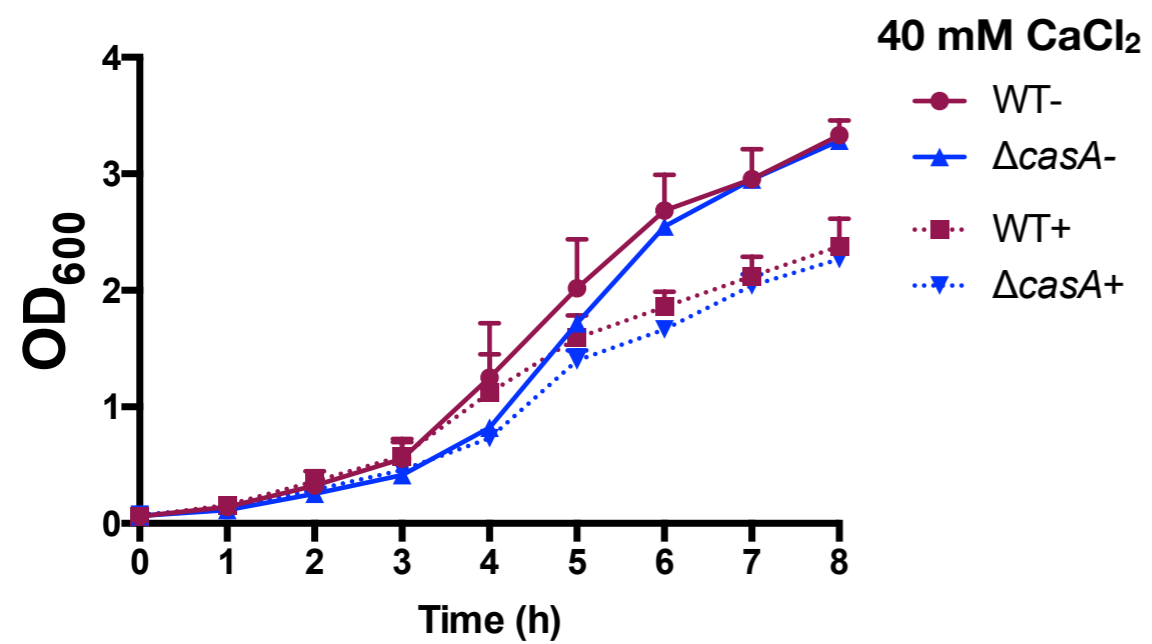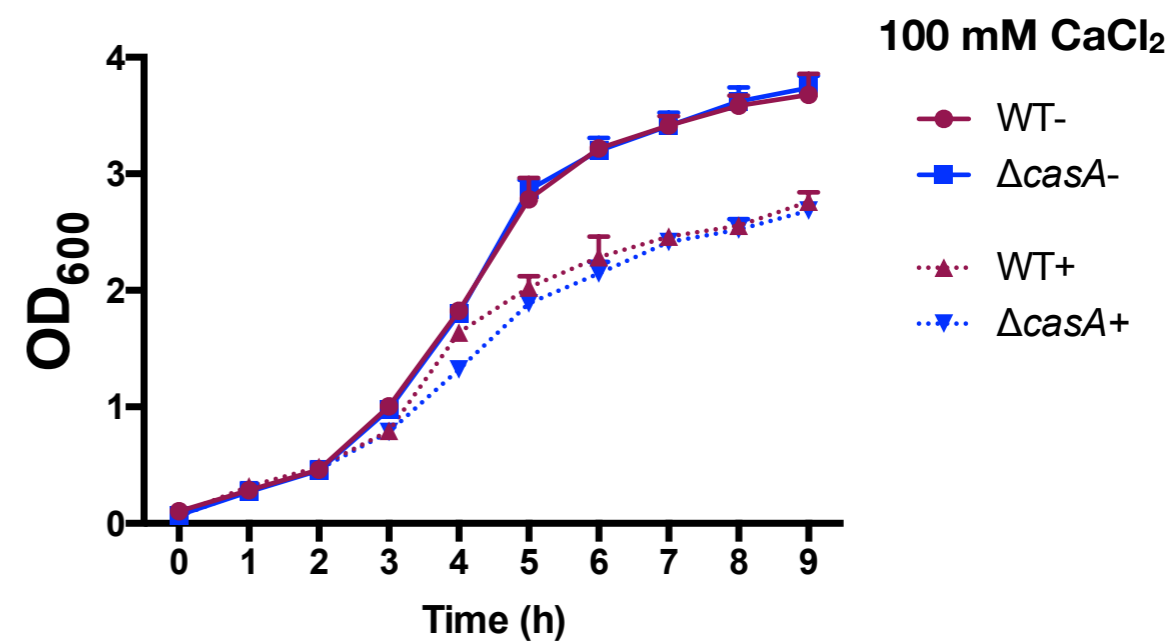

Supplement: FIG S1 [file mbio.02573-21-sf001.pdf]

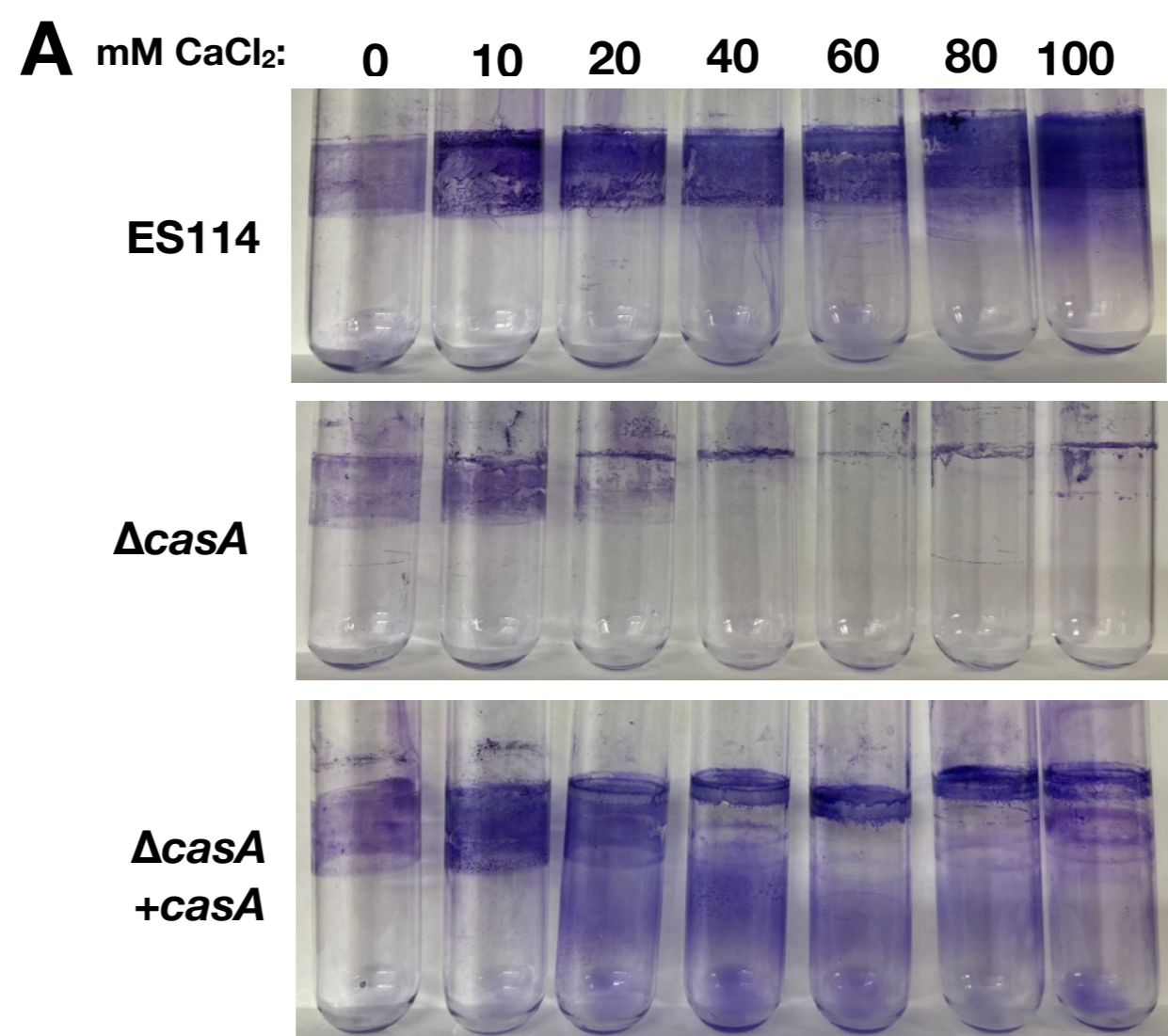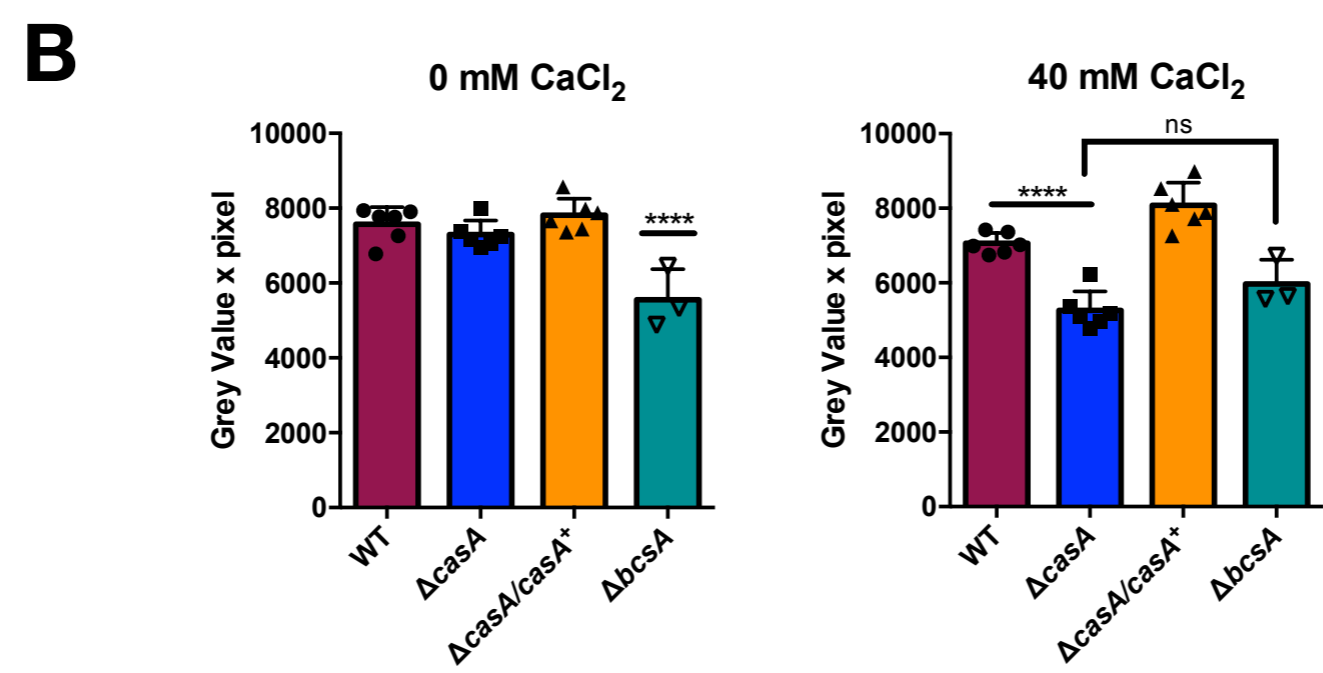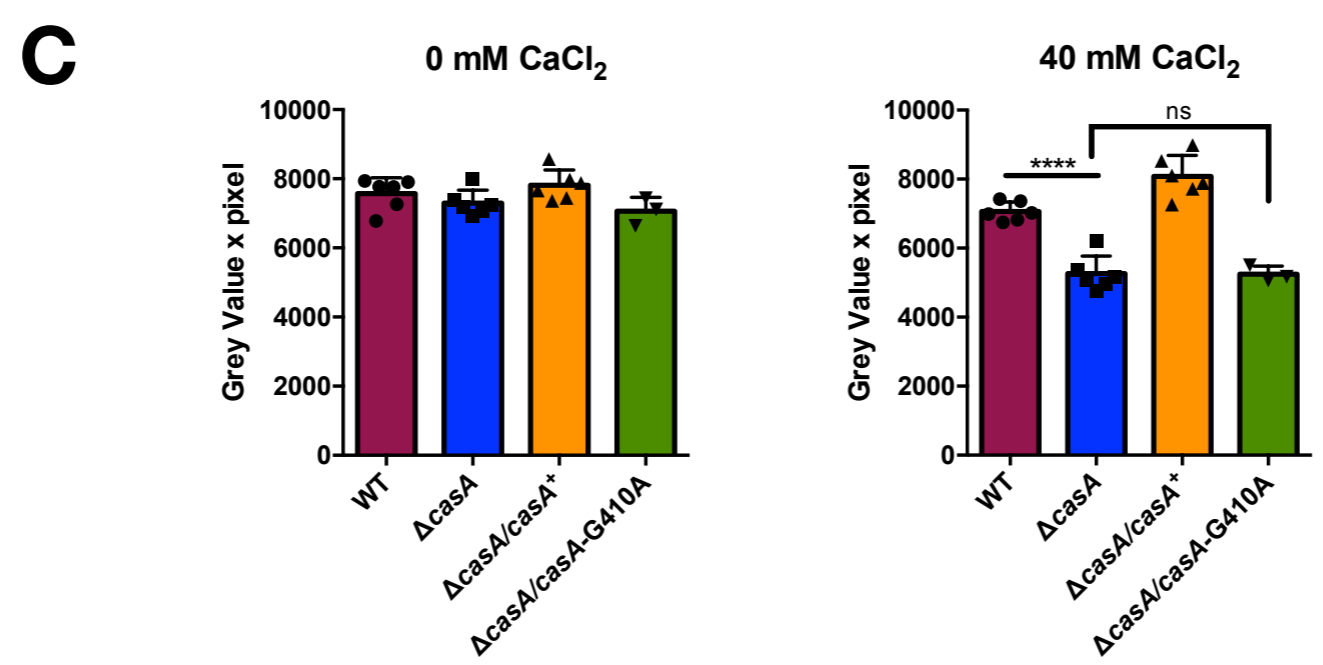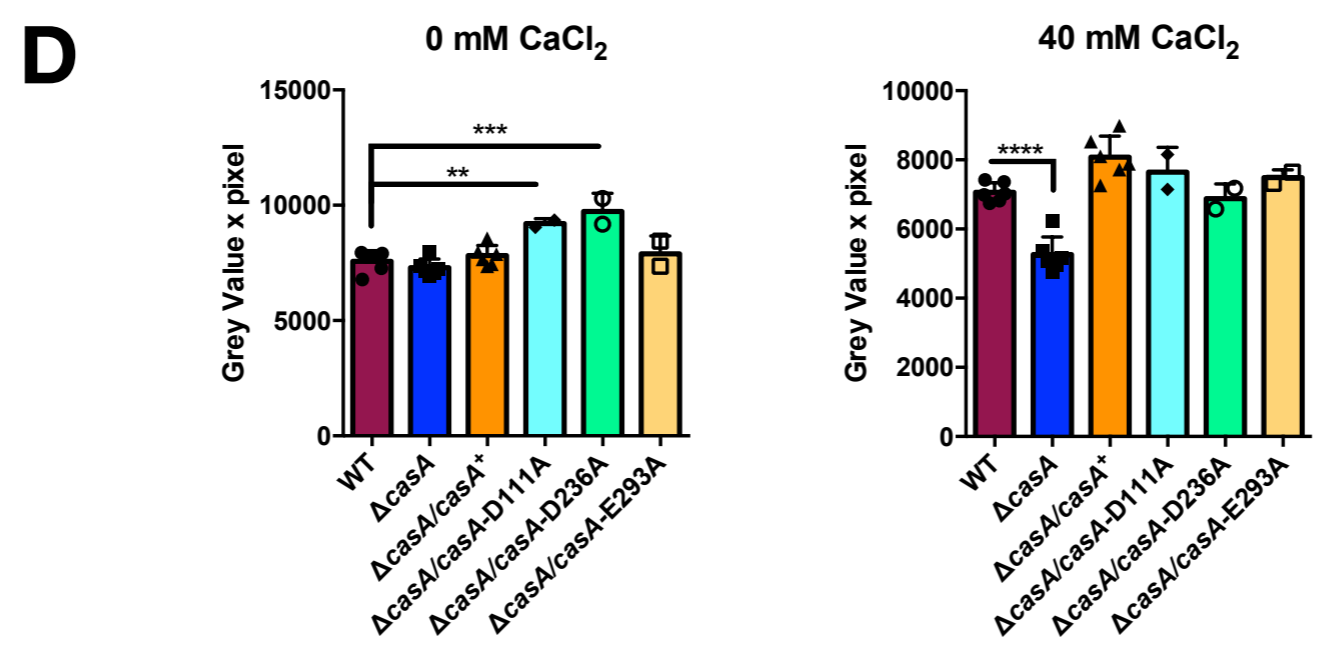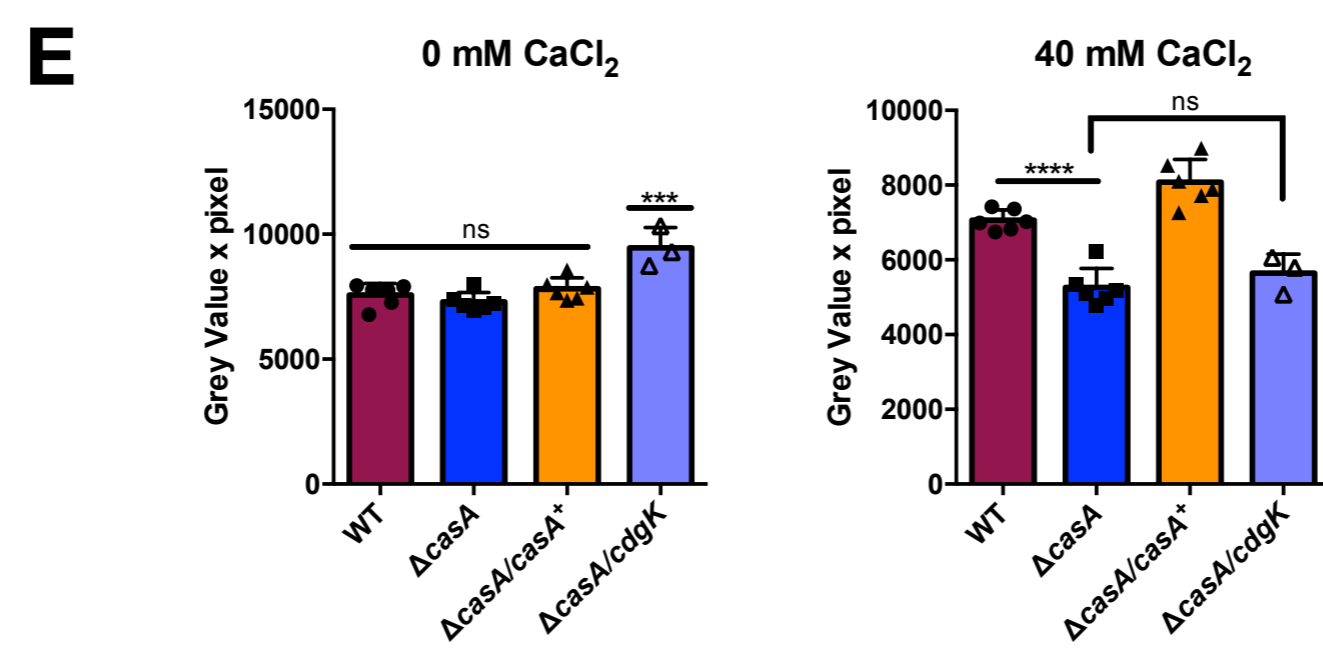

Supplement: FIG S2 [file mbio.02573-21-sf002.pdf]

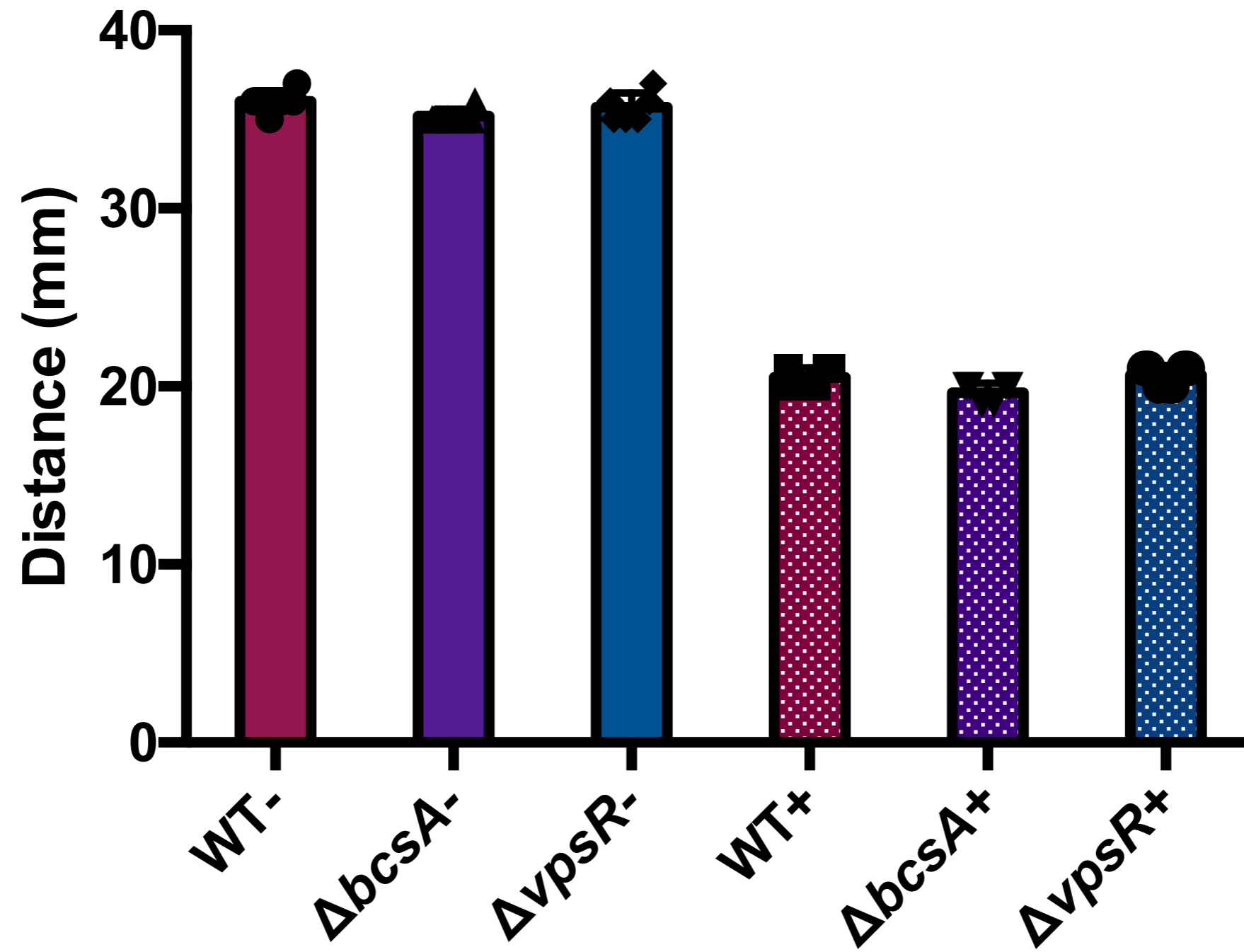

Supplement: FIG S3 [file mbio.02573-21-sf003.pdf]

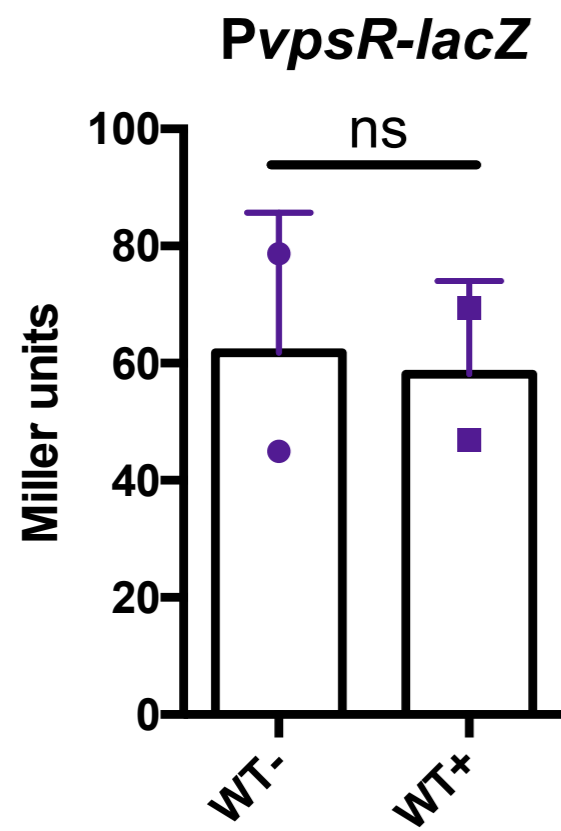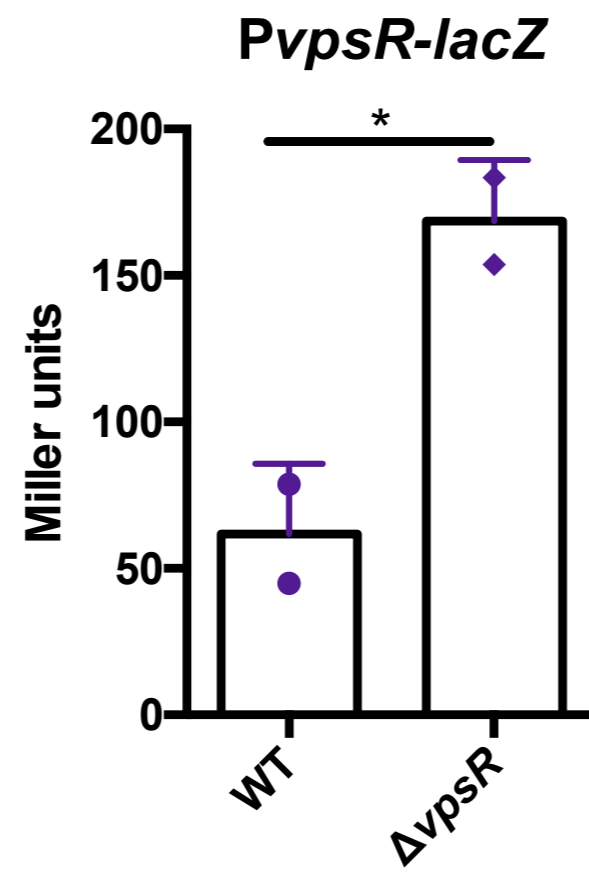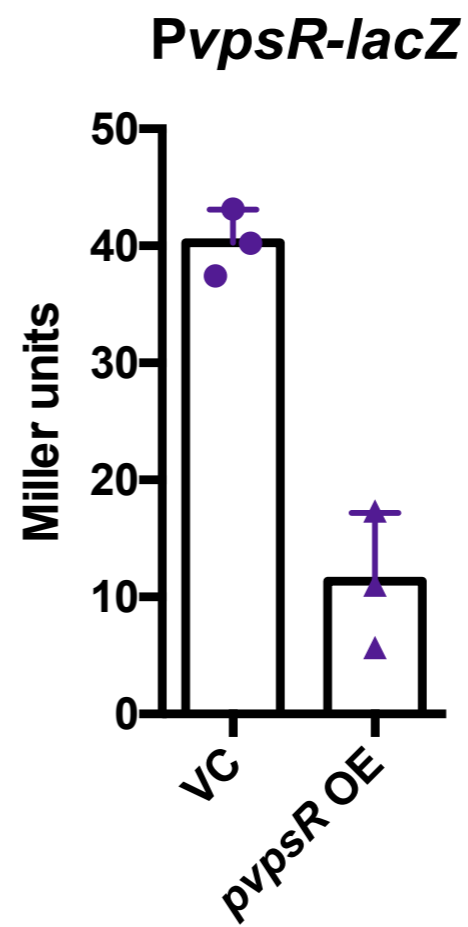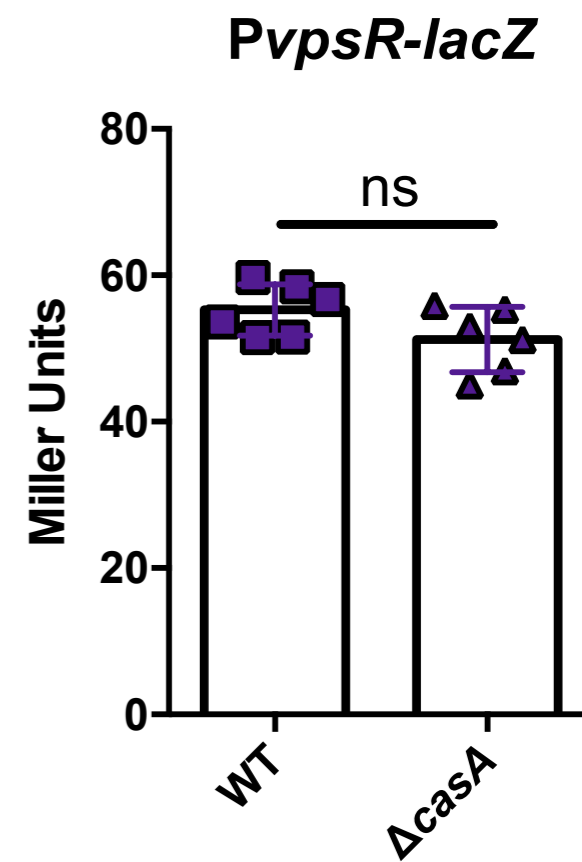

Supplement: FIG S4 [file mbio.02573-21-sf004.pdf]

**A**

~55kD

*casA*-HA  
*casA*-G410A-HA  
*casA*-D111A-HA  
*casA*-D236A-HA  
*casA*-E293A-HA  
*cdgK*-HA  
*casA*-G231A-HA

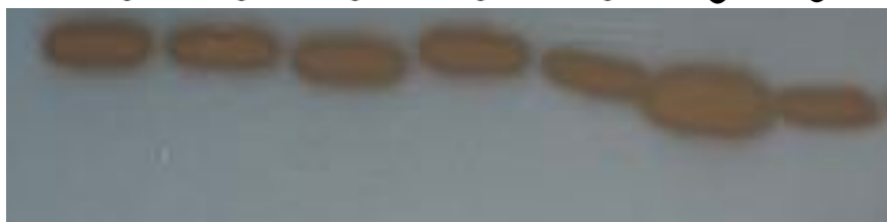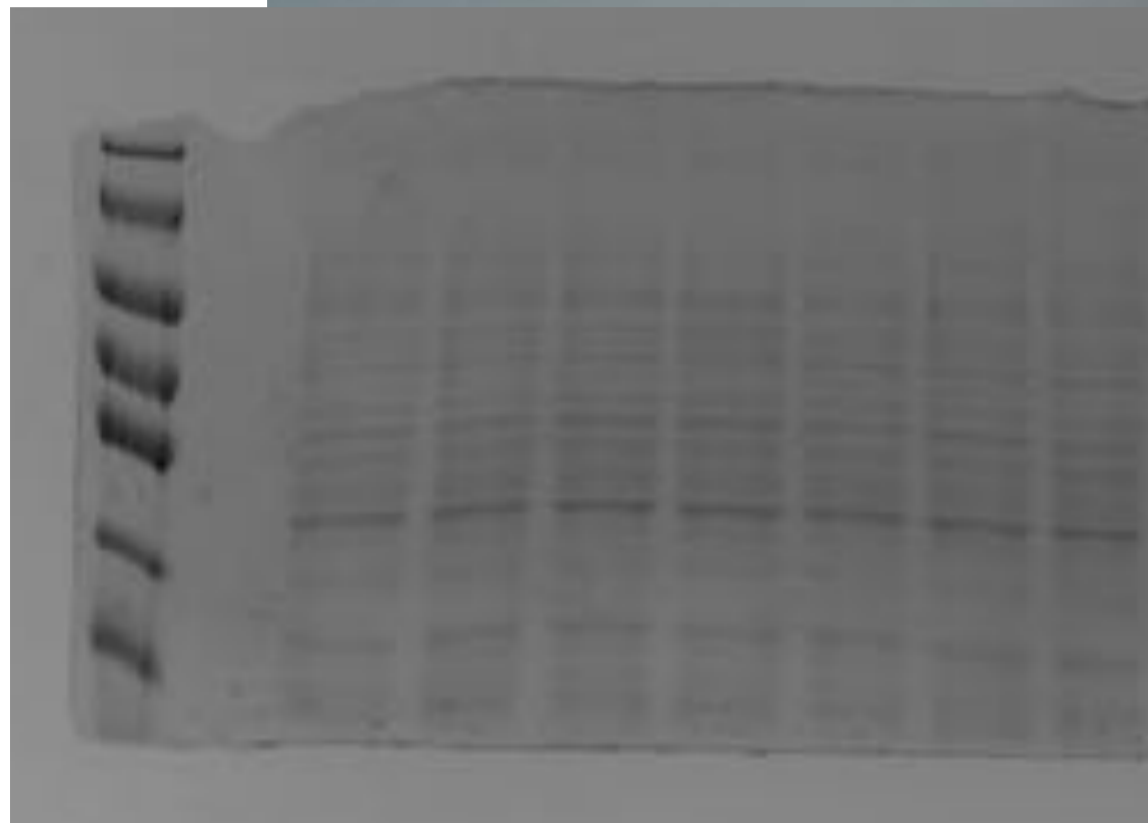**B**CaCl<sub>2</sub>:

0 10 20 40

~55kD

*cdgK*-HA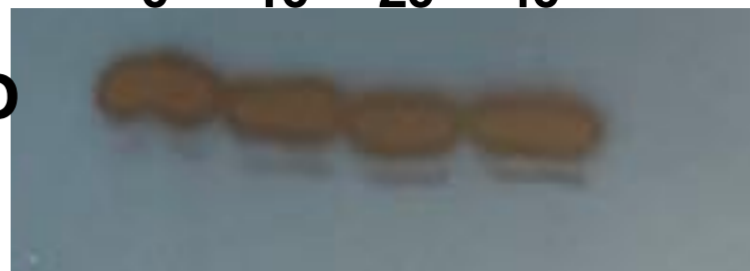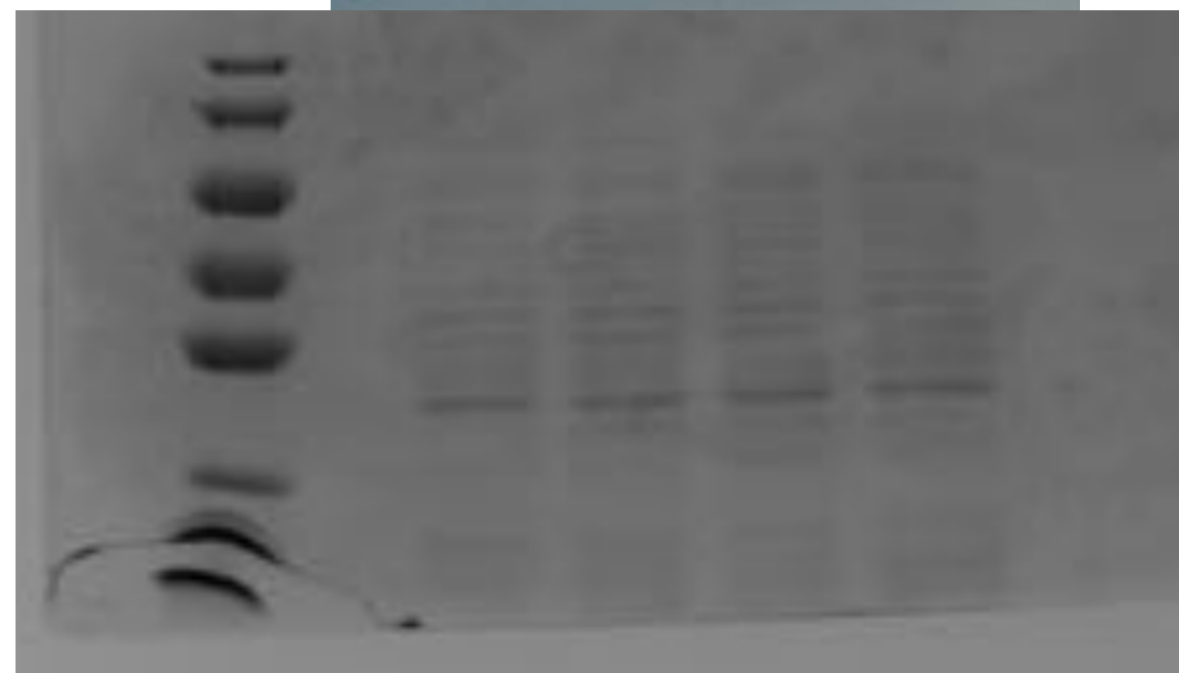**C**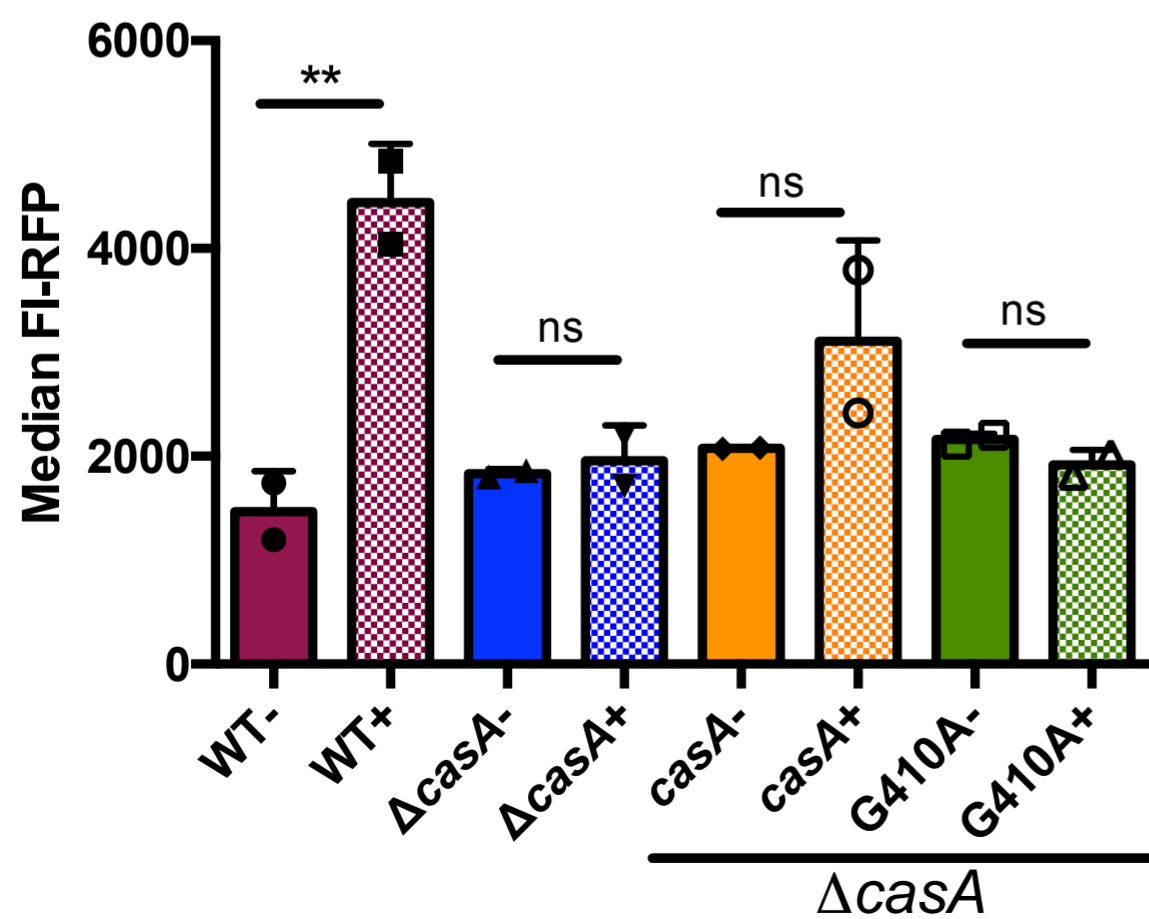**D**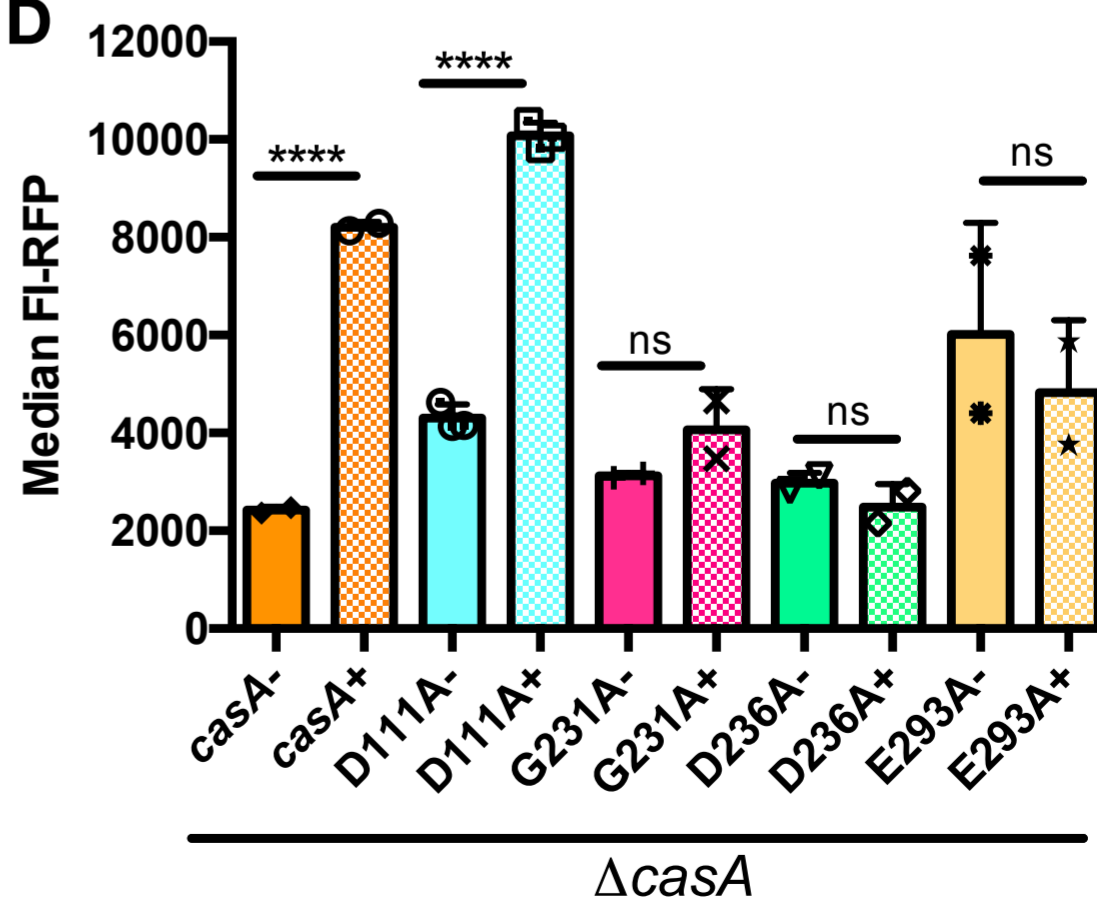

Supplement: FIG S5 [file mbio.02573-21-sf005.pdf]

T = 4 h

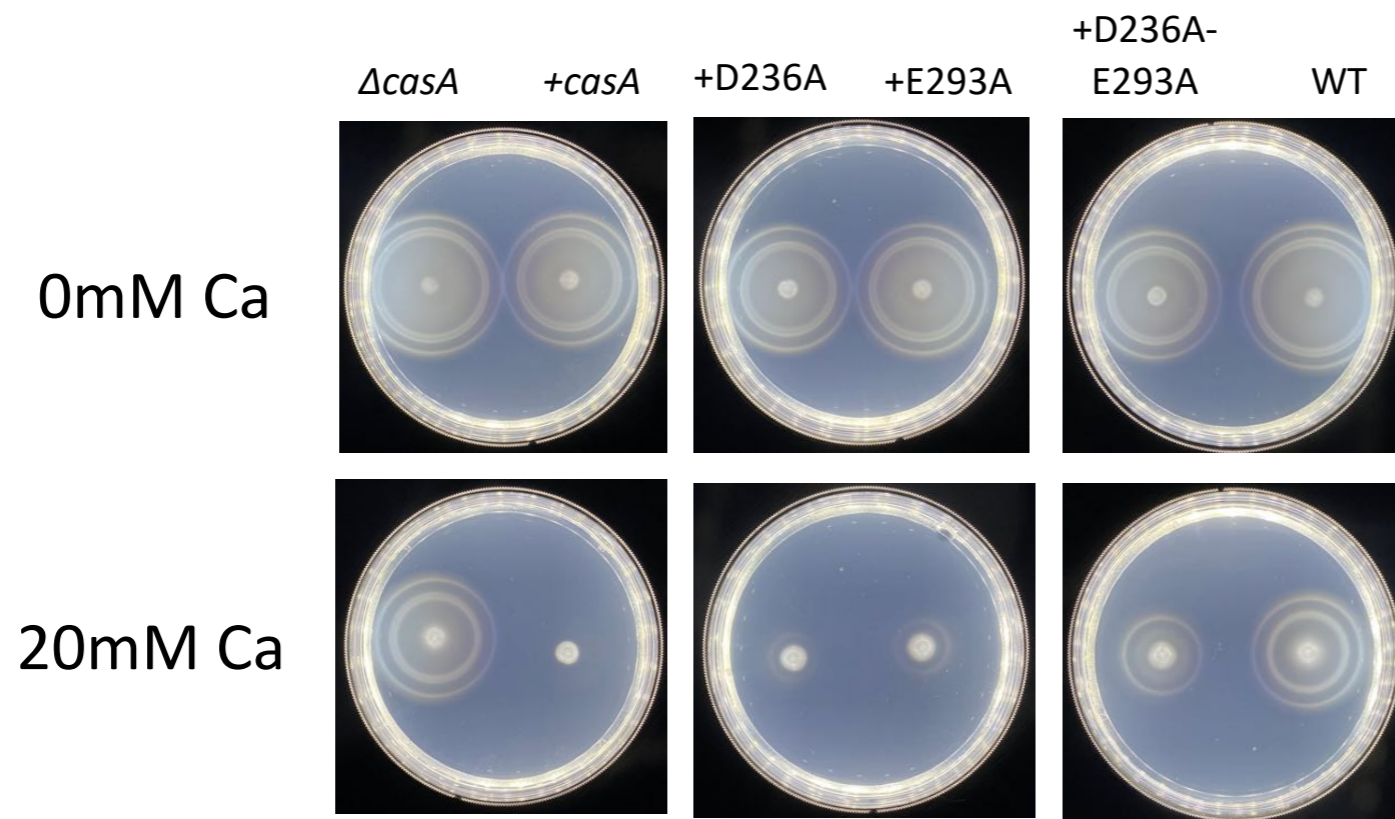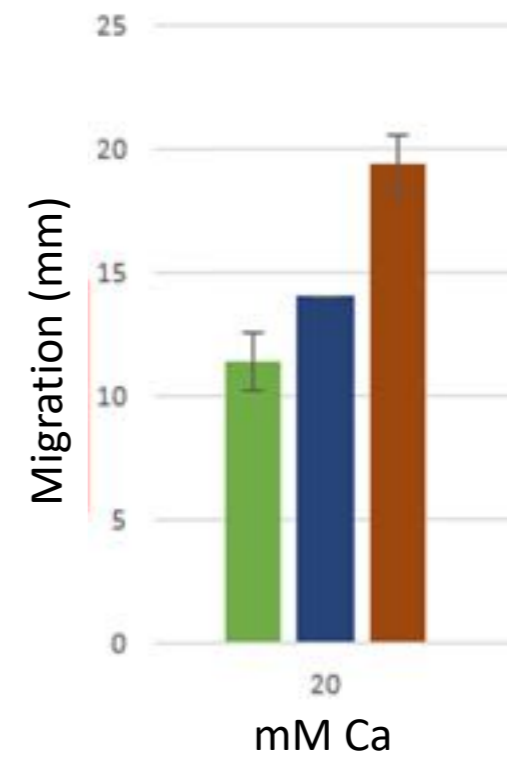

Supplement: FIG S7 [file mbio.02573-21-sf007.pdf]

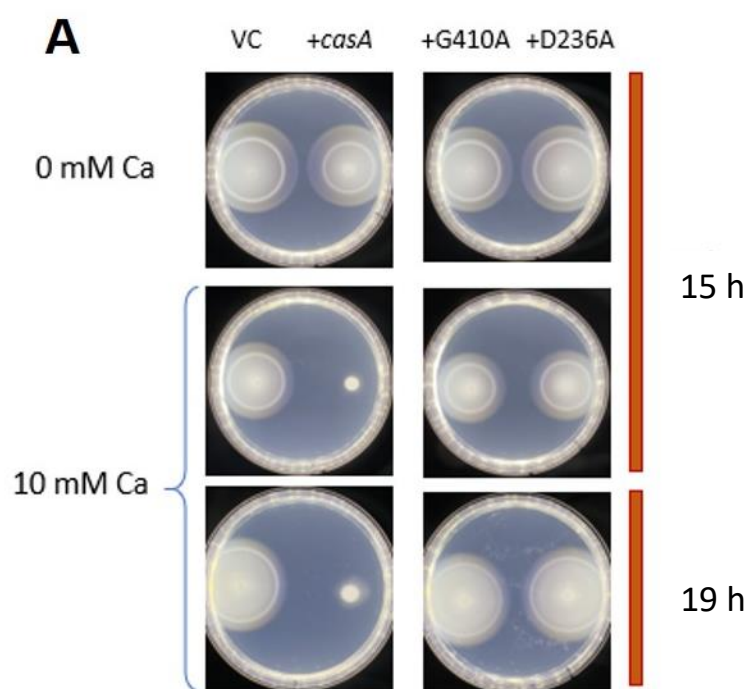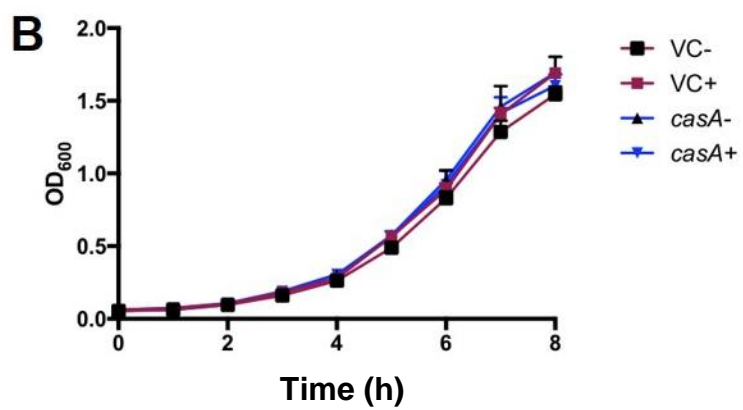

Supplement: FIG S8 [file mbio.02573-21-sf008.pdf]

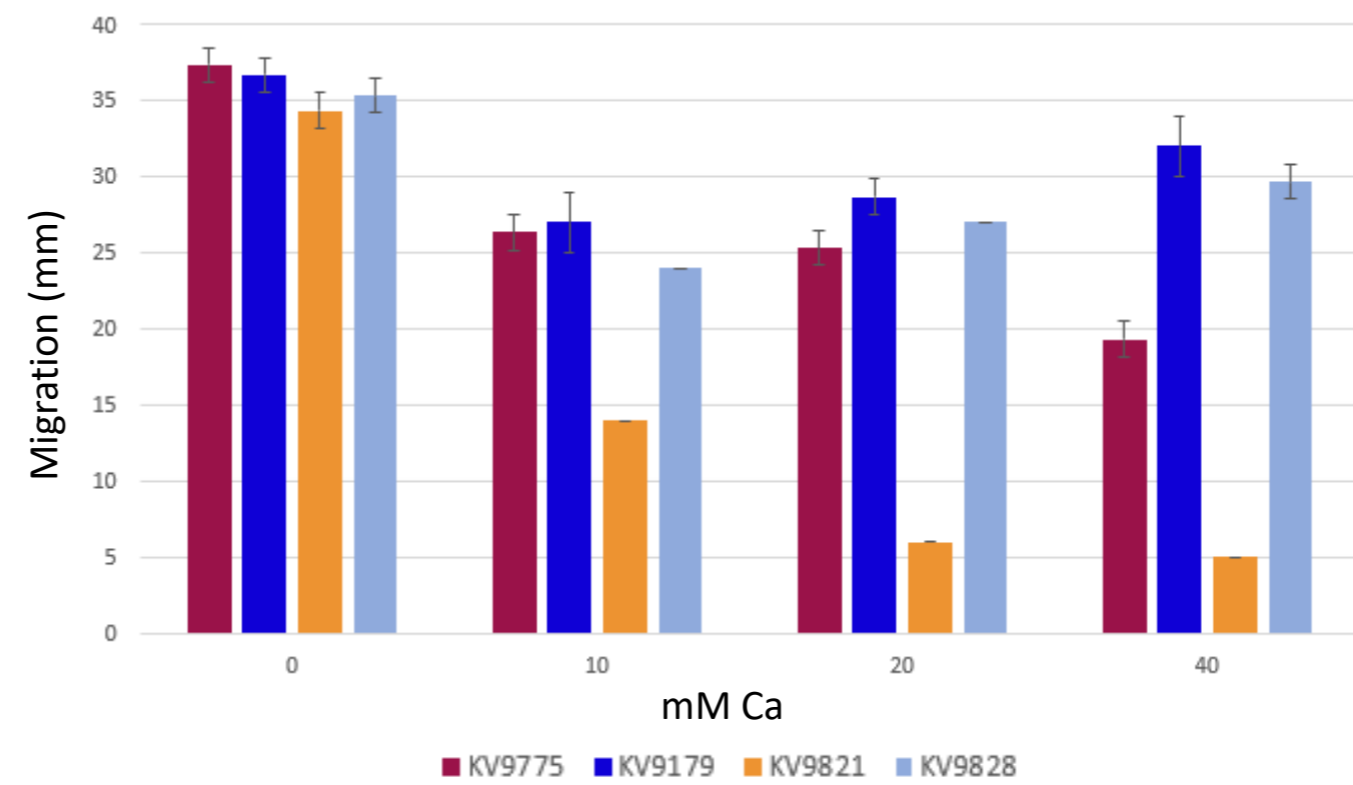

Supplement: FIG S9 [file mbio.02573-21-sf009.pdf]
