# Supplementary material for: Calcium-Responsive Diguanylate Cyclase CasA Drives Cellulose-Dependent Biofilm Formation and Inhibits Motility in Vibrio fischeri
Source: mBio. 2021 Nov 9;12(6):e02573-21. doi: 10.1128/mBio.02573-21 (PMC8576532; doi:10.1128/mBio.02573-21)
Supplement: FIG S6 [file mbio.02573-21-sf006.pdf]

A

```
V. fischeri             ---MPKFNLKHIFLIPTLFLVLIVGFILNNHMNSVQRQVNREYDRIINSLQRSIKVIISL    57
V. cholerae             --MNQRMSLTWIICAPLFIVFVLAASVFQQYLYDLNQEIDDAYADIQQQLERAEKVVTAL    58
V. sonorensis           MKTERHFSLAAVFFIPGVVTVILLGLIVKNYFDSVARDVSEEYQRIETSIERATKILTAL    60
V. crassostreae         MKVNRHFSLTFIFGFPAVIATMLLGLIGKNHFDSVKKDIDSEFHRIEEVFKRTTKVVTAL    60
V. coralliirubri        MKVNRHFSLTFVFGFPAVIAAMLLGLIGKNHFDLVKKDISSEFQRIEEVFKRTTKVVTAL    60
V. splendidus           MKVNRHFSLTFVFGFPAVIAAMLLGLIGKNHFDAVEKDISSEFHRIEDVFKRTTKVVTAL    60
                             ::.*  ::  *..  ::  . : ::::  : :::.  :  *   ::*: *:: :*

V. fischeri             DYNISQLYKQNQGSFYNHNFEIKKDSGLCVIRPSENLDAVMA-EQDISVAEGRLDYSIAG    116
V. cholerae             DYTFTHNQRPAENILFKHSARV--VANVCQIKPIDGLVLAQGLSSSFAVPKLDLSYMLIG    116
V. sonorensis           DYSFSNYYKSGTALLLEHNRTA--EDGLCRMWPIDALLLSEGKNQ--AIPAVDISYMLVG    116
V. crassostreae         DYSFSNYYKSGNPLFLDHNKQV--VDGLCQIWPIDVLLLADGKTS--DIPSVDIDYMLVG    116
V. coralliirubri        DYSFSNYYKSGNPLFLDHNKQV--VDGLCQIWPIDVLLLADGKTS--DIPSVDIDYMLVG    116
V. splendidus           DYSFSNYYKSGNPLFLDHNKHV--VDGLCQIWPIDVLLLADGKTS--DIPSVDIDYMLIG    116
                        **.:::  :     : .*.       .:* : * : *    .  .   :    :.* : *

V. fischeri             KKDLCDPSSEVYEIASKKVSLAPVISFIHDYESYLHGIYFISKYNYIISSPKEIAENLSQ    176
V. cholerae             DASLCDSKPSHDPSLQIKLSMAPILSFLHDMDEYIYGVHYVDSSGYIISSPDTISEKVTY    176
V. sonorensis           DKSLCDPNSPLYKRVSSKVSLAPILSFLHDLDDYVLGMHYIDKSGYVMSSPETLAKNINK    176
V. crassostreae         QESLCSETSDSYKSASEKIALAPILSFLSQLDEYHDGVHFIDTSGYVISSPEDFAKGLSK    176
V. coralliirubri        EESLCSESSDSYKSASKKIALAPILSFLSQLDEFHSGVHFIDKRGYVISSPEGFAKKLSK    176
V. splendidus           QESLCSESSDSYKSASEKIALAPILSFLSQLDEYHAGVHFIDTSGYVISSPEGFAKGLSK    176
                        . .**. .       . *:::**::**: : :.:  *::::.. .*::***. ::: :.

V. fischeri             K-TLDVIYSRPYWKNSMKNDSEKYITFTPPYTDAFFD-GLEVLTFSTPIYYKGIFKGVLV    234
V. cholerae             QQIKDFISESPLWSHAIAAPN--MIAVDGPYRSVIREQHEWLLTLILPVYRDADYQGLVA    234
V. sonorensis           S-LLETVKARPVWHITINNRE--TITIAGPANVY--SLSDRVVTMTVPIYFQDELQGIVS    231
V. crassostreae         E-LLSTIKSRPYWQKTANNPD--KLTLSGPGYRF--DSLDRMISMTIPVFHKGVHQGMLS    231
V. coralliirubri        E-LLSTIKSRPYWQKTANNPE--KLTLTGPAYRF--ESLDRMISMTIPVFHQGVHQGMLS    231
V. splendidus           E-LLSTIKSRPYWQKTANNPD--QLTLSGPGYRF--DSLDRIISMTIPVFHKGVHQGMLS    231
                        .   . :    * *  :    .   ::.  *      .    ::::  *:: .   :*::

V. fischeri             IDLSVEKLLRSS-NEISQHIQLLNSDEYKRFEQYRFMQPVNLDFTDFTYFLYYKTSIKEE    293
V. cholerae             VDIGLRELLSRM-PHLASRFDMIDLNEMAIPTFAYRPHKLTSEYADYHQVVFYKLDIQSE    293
V. sonorensis           LDLDIDALLSTN-GKLASPIHFSSDEPQLTPATARWIYPLKMEGVKFHHHLYYQFEWQPQ    290
V. crassostreae         VDINAGRLLENSNEHLAGRIDIIDTTRSAPIDSAAFYHEINLEGVASHHAMYYELDIAKE    291
V. coralliirubri        VDIDADKLLANSNEHLAGRIDIIDTTLATPVDSAAFYHELKLDGVASHHAMYYELDIAKE    291
V. splendidus           VDINADKLLANSNEHLAGRIDIIDTTLATPVDSAAFYHEIKLDGVSSHHAMYYELDIAKE    291
                        :*:.   **     .::  :.: .                :. :  .     ::*:  .   :

V. fischeri             LKGFLVHDSNSLILTLVMYILSLLSMFYYQSRVSQKYYRDLAKQDPMTGLYNRRGFEMSL    353
V. cholerae             LSNFLTEKSGNLLVVALVYLFLTGVLIYLNTRWDRQHYAQLAARDPMTGLLNRRGMESFL    353
V. sonorensis           VQHFFALESDSLAVIASLYVMSVFVLFYINTHVEKSYFRELSAKDPMTGLLNRRGLEAFL    350
V. crassostreae         IEHFFVYEKDSLIVAIIVYLFSVTIFFYVNSTIERGYFKDLAAKDPMTGLLNRRGLEAFW    351
V. coralliirubri        VEHFFVYEKDSLIVAIIVYLFSVTIFFYVNSNIERGYFKDLAAKDPMTGLLNRRGLEAFW    351
V. splendidus           VEHFFVYEKDSLIVAIIVYLFSVTIFFYVNSNIERGYFKDLAAKDPMTGLLNRRGLEAFW    351
                        :. *:. ....* :   :*::    ::* ::  .: :: :*: :****** ****:*

V. fischeri             QDRVVKKYVGFAIYDIDDFKQINDVFGHDVGDEAIKYVARMLNKSVRDSDIVSRFGGEEF    413
V. cholerae             KGKRHSQYLAIAVLDIDDFKQINDAYGHDMGDRVICYIGEQIENHIRSSDAVARFGGEEF    413
V. sonorensis           GNAQHGNYLAIAIFDIDNFKSINDTWGHDVGDDVIRHIGKELEKNLRSNDSVARFGGEEF    410
V. crassostreae         RSVEHDQLFALTVFDIDDFKSINDTYGHDKGDDVIRYMSRQIGNSVRSSDVAARFGGEEF    411
V. coralliirubri        RSVEHDQLFALTVFDIDDFKSINDTYGHDKGDDVIRYMSRQINNSIRSSDVAARFGGEEF    411
V. splendidus           RSVEHDQLFALTVFDIDDFKSINDTYGHDKGDDVIRYMSRQISNSIRSSDVAARFGGEEF    411
                         .    : ..::: ***:**.***.:*** ** .* ::.. : : :*..* .:*******

V. fischeri             VICINAESRNSLESVCERVRKSIQDSSGKVVKGGFTVSGGVTVIDS-HQEFSFEHVIKKA    472
V. cholerae             VVYVTAKEKEQITRIMQRIFDAVCRESPLILEPGFTISGGIEVVES-TTDRSFEDLFKAA    472
V. sonorensis           VIYMTGDAKDGLVASMQRVRSAIGESSFKVLEKGFTLSGGIEVAKS-EAGWDFETMFKAA    469
V. crassostreae         VVYMRGEDRETLMRTLERVKNAICSTSADIIPNGFTVSGGVCIVETEQSKLNFDEIFKYA    471
V. coralliirubri        VVYMKGEDRETLMRTLERVKNAICSTSADIIPNGFTVSGGVCIVETEQSKLNFDEIFKYA    471
V. splendidus           VVYMKGEDRETLMRTLERVKNAICSTSADIIPNGFTVSGGICLVETEQSKLNFDEIFKYA    471
                        *: : .. :: :    :*: .::   *  ::  ***:***: : .:     .*: ::* *

V. fischeri             DALLYKAKQDGKNRVYFS      490
V. cholerae             DEKLYVAKTSGKNQLVY-      489
V. sonorensis           DEKLYLAKNQGKDQLVS-      486
V. crassostreae         DEKLYVAKTTGKDRLEF-      488
V. coralliirubri        DEKLYVAKTTGKDRLEF-      488
V. splendidus           DEKLYVAKTTGKDRLEF-      488
                        *  ** **  **:::
```

B

```
CasA          -MPKFNLKHIFLIPTLFLVLIVGFILNNHMNSVQRQVNREYDRIINSLQRSIKVIISLDY 59
CdgK          MNQRMSLTWIICAPLFIVFVLAASVFQQYLYDLNQEIDDAYADIQQQLERAEKVVTALDY 60
                 ::.*. *:  * :::.::.. :::::: .::::::  *  * :.*:*: **: :***

CasA          NISQLYKQNQGSFYNHNFEIKKDSGLCVIRPSENLDAVM-AEQDISVAEGRLDYSIAGKK 118
CdgK          TFTHNQRPAENILFKHSAR--VVANVCQIKPIDGLVLAQGLSSSFAVPKLDLSYMLIGDA 118
              .:::  :  :. :::*. .    :.:* *:* :.*  .   ...::* :  *.* : *.

CasA          DLCDPSSEVYEIASKKVSLAPVISFIHDYESYLHGIYFISKYNYIISSPKEIAENLSQKT 178
CdgK          SLCDSKPSHDPSLQIKLSMAPILSFLHDMDEYIYGVHYVDSSGYIISSPDTISEKVTYQQ 178
              .*** . .     . *:*:**::**:** :.*::*::::.. .******. *:*::: :

CasA          -LDVIYSRPYWKNSMKNDSEKYITFTPPYTDAFFDGL-EVLTFSTPIYYKGIFKGVLVID 236
CdgK          IKDFISESPLWSHAIAAP--NMIAVDGPYRSVIREQHEWLLTLILPVYRDADYQGLVAVD 236
                *.* . * *.:::      : *:.  ** ..: :    :**:  *:* .. ::*::.:*

CasA          LSVEKLLRSSNEISQHIQLLNSDEYKRFEQYRFMQPVNLDFTDFTYFLYYKTSIKEELKG 296
CdgK          IGLRELLSRMPHLASRFDMIDLNEMAIPTFAYRPHKLTSEYADYHQVVFYKLDIQSELSN 296
              :.:.:**    .::.:::::: :*           : :. :::*:  .::** .*:.**..

CasA          FLVHDSNSLILTLVMYILSLLSMFYYQSRVSQKYYRDLAKQDPMTGLYNRRGFEMSLQDR 356
CdgK          FLTEKSGNLLVVALVYLFLTGVLIYLNTRWDRQHYAQLAARDPMTGLLNRRGMESFLKGK 356
              **...*..*::. ::*::    ::* ::* .:::* :** :****** ****:*  *:.:

CasA          VVKKYVGFAIYDIDDFKQINDVFGHDVGDEAIKYVARMLNKSVRDSDIVSRFGGEEFVIC 416
CdgK          RHSQYLAIAVLDIDDFKQINDAYGHDMGDRVICYIGEQIENHIRSSDAVARFGGEEFVVY 416
                .:*:.:*: **********.:***:**..* *:.. ::: :*.** *:********:

CasA          INAESRNSLESVCERVRKSIQDSSGKVVKGGFTVSGGVTVIDSHQEFSFEHVIKKADALL 476
CdgK          VTAKEKEQITRIMQRIFDAVCRESPLILEPGFTISGGIEVVESTTDRSFEDLFKAADEKL 476
              :.*:.::.:  : :*: .::   .*  ::: ***:***: *::*  : ***.::* **  *

CasA          YKAKQDGKNRVYFS 490
CdgK          YVAKTSGKNQLVY- 489
              * ** .***:: :
```
