# Supplementary material for: Calcium-Responsive Diguanylate Cyclase CasA Drives Cellulose-Dependent Biofilm Formation and Inhibits Motility in Vibrio fischeri
Source: mBio. 2021 Nov 9;12(6):e02573-21. doi: 10.1128/mBio.02573-21 (PMC8576532; doi:10.1128/mBio.02573-21)
Supplement: TEXT S1 [file mbio.02573-21-s0001.pdf]

Supplemental Methods:

Western immunoblotting. *V. fischeri* cells were grown overnight in LBS, and cultures were normalized to an OD_{600} of 1. One-ml aliquots were pelleted and lysed in 200 μl 2X sample buffer (4% SDS, 40 mM Tris, pH 6.3, 10% glycerol). Samples were loaded in two separate gels in tandem, and separated by SDS-PAGE (8% acrylamide). One gel was stained with Coomassie blue (40% MeOH, 10% acetic acid, 0.1% Coomassie Blue R250) as a loading control, destained (40% MeOH, 10% acetic acid) and imaged using FluorChem (ProteinSimple). The second gel was transferred to a polyvinylidene difluoride (PVDF) membrane. After blocking with dry milk in PBS-T (137 mM NaCl, 2.7 mM KCl, 4.3 mM Na_2HPO_4, 1.47 mM KH_2PO_4, and 0.05% Tween 20), the membrane was treated with rabbit anti-HA antibody (Sigma-Aldrich), followed by exposure to a secondary antibody, goat anti-rabbit IgG antibody (Fisher Scientific) conjugated to horseradish peroxidase. Finally, to visualize HA-tagged proteins, the membrane was incubated with Super- Signal West Pico plus chemiluminescent substrate (Thermo Fischer Scientific, Rockford, IL) and exposed to autoradiography film (Dot Scientific), which was developed in an autoprocessor.

Growth curves. Single colonies were inoculated in triplicate in either TBS or TB broth for *V. fischeri* or *E. coli*, respectively, and grown overnight shaking at 28˚C. Strains were subcultured into 125 ml baffled flasks containing 30 mL broth supplemented with calcium chloride as indicated, to a starting OD_{600} of 0.05. Samples were collected at 1-hour intervals for eight hours and OD_{600} was measured for each sample immediately after collection.

Congo red image quantification. Overnight cultures were normalized to an OD_{600} of 0.2, then 10 μl aliquots were spotted and grown at 24°C for 24 hours. Image quantification was performed by

determining grey value per pixel of each spot. Data were analyzed using one-way ANOVA in GraphPad, Prism 6.
